# Three new C-27-carboxylated-lupane-triterpenoid derivatives from *Potentilla* discolor Bunge and their *in vitro* antitumor activities

**DOI:** 10.1371/journal.pone.0175502

**Published:** 2017-04-07

**Authors:** Jing Zhang, Chao Liu, Ri-Zhen Huang, Hui-Feng Chen, Zhi-Xin Liao, Jin-Yue Sun, Xue-Kui Xia, Feng-Xiang Wang

**Affiliations:** 1 Department of Pharmaceutical Engineering, School of Chemistry and Chemical Engineering, Southeast University, Nanjing, P. R. China; 2 Institute of Agro-Food Science and Technology/Key Laboratory of Agro-Products Processing Technology of Shandong Province, Shandong Academy of Agricultural Sciences, Jinan, P. R. China; 3 Biotechnology Center, Shandong Academy of Sciences, Jinan, P. R. China; 4 Institute of Shandong River Wetlands, Laiwu, P. R. China; Duke University School of Medicine, UNITED STATES

## Abstract

Three new lupane-triterpenoids (**1**–**3**) along with six known compounds (**4**–**9**) were isolated from the ethanolic extract of whole plant of *Potentilla discolor* Bunge. The structures of Compounds **1**–**3** were established by extensive 1D and 2D NMR together with other spectrum analysis, indicating that their C-27 positions were highly oxygenated, which were rarely found in nature. Their *in vitro* anti-proliferative activities against HepG-2, MCF-7 and T-84 cell lines were evaluated by Cell Counting Kit-8 (CCK-8) assay, and the results showed different activities for three cell lines with IC_50_ values ranging from 17.84 to 40.64 μM. In addition, the results from Hoechst 33258 and AO/EB staining as well as annexinV-FITC assays exhibited Compound **1** caused a markedly increased HepG-2 cellular apoptosis in a dose-dependent manner. The further mechanisms of Compound **1**-induced cellular apoptosis were confirmed that **1** induced the production of ROS and the alteration of pro- and anti-apoptotic proteins, which led to the dysfunction of mitochondria and activation of caspase-9 and caspase-3 and finally caused cellular apoptosis. These results would be useful in search for new potential antitumor agents and for developing semisynthetic lupane-triterpenoid derivatives with high antitumor activity.

## Introduction

*Potentilla* is one of a hundred genera in the family Rosaceae, subfamily Rosoideae, tribe Potentilleae [1, 2], which contains about 700 species. They are widespread in temperate, arctic and Alpine zones of the Northern hemisphere. *Potentilla* species have been used in traditional medicine of different cultures by Asian, European and American for a long time [3–8]. In addition, several pharmacopoeias include monographs on *Potentilla* species, e.g. European Pharmacopoeia 9.0, 2017 [9] and Chinese Pharmacopoeia 2015 [10].

*Potentilla discolor* Bunge (PD), one of the species of *Potentilla*, is a perennial herb which abundantly distribute in China, especially in Liaoning, Anhui, Shanxi and Shandong Provinces. This plant widely grows in sparse forests, meadows, valleys and ravines, whose roots are robust, enlarged and fusiform, flowering stems erect, and pinnate leaves are oblong with margin incised dentations [11]. This species has been used traditionally in folk medicine for the treatment of diarrhea, hepatitis, functional uterine hemorrhage and traumatic hemorrhage [12, 13]. In recent years, whole herbs of PD have also been used to treat type 2 diabetes in clinical research [14–16], moreover, some scientific evidences have confirmed the effectiveness of this plant as an anticancer agent [17].

The principal phytochemicals in PD have been reported to be triterpenoids and flavonoids, besides, hydrolysable tannins, sterols, and some aromatic acids have been isolated [16, 18–21]. The ethanolic extract of PD has shown significant inhibition on the proliferation of tumor cells and the induction of cellular apoptosis at a low concentration in previous literature [17], however, there were few phytochemistry researches which focused on antitumor activities. Furthermore, triterpenoids of different structural types have been shown to be antitumor agents in a large number of reports [22–26]. All these research basics prompted us to investigate the chemical constituents of PD. As a result, three new lupane-triterpenoids were obtained from its ethanolic extract, which were rarely found in nature due to their C-27 positions were highly oxygenated [27]. In addition, six other known compounds were also obtained. Moreover, the levels of antitumor activities for the three new compounds were examined. Then, we further investigated the detailed mechanism of apoptotic effects induced by the representative Compound **1**.

## Materials and methods

### General experimental procedures

Optical rotations were measured on a WZZ-2B spectropolarimeter. IR spectra were recorded on a NICOLET IR200 FT-IR spectrophotometer. NMR spectra were recorded on a Bruker Avance DRX-400 spectrometer at 400 MHz (^1^H) and 100 MHz (^13^C). Chemical shifts were expressed in *δ* (ppm) referring to TMS. HR-ESI-MS was carried out on an Agilent Technologies 6224 TOF LC-MS apparatus. Purity of compounds were determined by HPLC (Agilent 1200) on a column (Silgreen GH0525046C18A; 250 × 4.6 mml.D., S-5 μm, 12 nm). Apoptosis was discriminated with Beckman Coulter CytoFLEX flow cytometry.

### Plant materials

**Fig 1:**
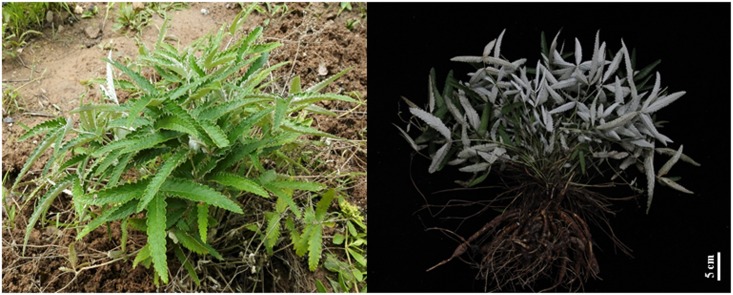
Morphological observation of *Potentilla dicolor* Bunge from Mountain Tai.

Whole herbs of PD (Fig 1) were collected in a private mountain land of Mountain Tai (36°16′N; 117°6′E; 1532.7 m a.s.l.) owned by a local peasant, Shandong, China, in August, 2015, and got his permission to conduct this activity. PD is not an endangered or protected species, which abundantly distributes in China and is a common medicine herb in local. Besides, our collection activities didn’t cause any negative impact on the local ecology. The species was identified by Dr. Chenggang Shan, Institute of Agro-Food Science and Technology, Shandong Academy of Agriculture Sciences, Jinan, China. A voucher specimen (No. 15–08–20) was deposited at nature medicine laboratory of Southeast University.

### Extraction and isolation

The dried plant (5 kg) was ground into powder and then extracted with 95% ethanol (20.0 L) by heating reflux for 4 × 3 hours using a reaction still. Following filtration and vacuum-concentration of the combined solution, it yielded a crude extract (478.8 g). 400g extract was then suspended in water (2000 mL) and extracted successively with petroleum ether (5 × 2000 mL), and EtOAc (5 × 2000 mL) to afford two organic fractions.

The extract of petroleum ether extract (50.0 g) was separated by column chromatography (CC) over silica gel, eluted with various petroleum ether-ethyl acetate gradients (50:1, 40:1, 30:1, 20:1, 15:1, 12:1, 10:1, 8:1, 6:1, 5:1, 4:1, 3:1, 2:1, 1:1, 0:1, 3.6 L each) to yield 7 subfractions (Fr.1-Fr.7). Fr.2–6 were firstly subjected to MCI gel CC (80% ethanol) to remove pigments. Fr. 3 (petroleum ether-ethyl acetate 6:1, ca. 3.1g) was chromatographed over silica gel eluted with petroleum ether-ethyl acetate (13:1–3:1) to furnish **5** (50.1 mg). Fr.4 (petroleum ether-ethyl acetate 5:1, ca. 0.5 g) was chromatographed over Sephadex LH-20 eluting with dichloromethane-methanol (1:1) to yield **3** (10.4 mg). Fr.5 (petroleum ether-ethyl acetate 4:1–3:1, ca. 3.3 g) was submitted to silica gel CC eluted with petroleum ether-ethyl acetate (12:1–2:1) to yield Compound **1** (30.1 mg), **2** (12.3 mg).

The extract of EtOAc (32.8 g) was fractionated by column chromatography over silica gel eluting with petroleum ether followed by increasing concentration of ethyl acetate to yield 4 fractions (Fr.1-Fr.4). Fr.2 (petroleum ether-ethyl acetate 4:1–3:1, ca. 6.2g) was purified by silica gel CC eluted by petroleum ether-ethyl acetate (10:1–1:1) to give Compound **6** (9.4 mg), **9** (25.0mg). Fr.3 (petroleum ether-ethyl acetate 2:1, ca. 8.4g) was firstly fractioned to two subfractions (3a and 3b) by column chromatography over silica gel eluting with gradient elution (petroleum ether-ethyl acetate 5:1–1:1). Compound **7** (12.0mg) was derived from sub. 3a (3.2 g) by repeated CC with petroleum ether-ethyl acetate (2:1). The separation of sub.3b (2.3 g) separated over Sephadex LH-20 eluting with dichloromethane-methanol (1:1) to yield **8** (21.1 mg). Compound **4** (12.8 mg) was gained from Fr.4 (9.7 g, petroleum ether-ethyl acetate 1:1) which was subjected to CC over silica gel eluted with petroleum ether-ethyl acetate (4:1–1:2).

### Compound characterization

3*α*-hydroxy-19*α*-hydrogen-29-aldehyde-27-lupanoic acid (**1**): white amorphous powder, mp 201–202°C, purity 98.35%. [*α*]20 D = -10.00 (c = 0.29, MeOH). IR (KBr, max, cm^-1^): 3640, 2949, 2862, 1718, 1237, 1215. ^1^H and ^13^C NMR (CHCl_3_) (see Table 1). HR-ESI-MS: 495.3558 [M + Na]^+^) ([C_30_H_48_O_4_+ Na]^+^, calc: 495.3553).

**Table 1:** ^1^H and ^13^C NMR data of 1–3 at 400 and 100 MHz in CDCl_3_ (*δ* in ppm; *J* in Hz).

Position	1		2		3	
	^1^H NMR	^13^C NMR	^1^H NMR	^13^C NMR	^1^H NMR	^13^C NMR
**1a**	1.25 (1H, m)	33.22	1.28 (1H, m)	33.22	1.14 (1H, m)	33.84
**1b**	1.45 (1H, m)		1.48 (1H, m)		1.45 (1H, m)	
**2a**	1.58 (1H, m)	25.05	1.58 (1H, m)	25.03	1.58 (1H, m)	25.06
**2b**	1.72 (1H, m)		1.72 (1H, m)		1.72 (1H, m)	
**3**	3.42 (1H, t)	76.19	3.4 (1H, t)	76.16	4.61 (1H, t)	78.04
**4**		37.56		38.78		37.48
**5**	1.22 (1H, m)	48.95	1.20 (1H, m)	48.39	1.22 (1H, m)	49.25
**6a**	1.31 (1H, m)	18.17	1.35 (1H, m)	18.13	1.31 (1H, m)	18.05
**6b**	1.41 (1H, m)		1.43 (1H, m)		1.41 (1H, m)	
**7a**	1.32 (1H, m)	37.38	1.35 (1H, m)	37.48	1.35 (1H, m)	37.22
**7b**	1.46 (1H, m)		1.40 (1H, m)		1.41 (1H, m)	
**8**		40.77		42.55		40.8
**9**	1.28 (1H, m)	49.93	1.61 (1H, m)	50.7	1.28 (1H, m)	50.19
**10**		37.5		37.54		37.45
**11a**	1.32 (1H, m)	20.49	1.31 (1H, m)	20.62	1.32 (1H, m)	21.34
**11b**	1.59 (1H, m)		1.62 (1H, m)		1.59 (1H, m)	
**12a**	1.64 (1H, m)	27.25	1.64 (1H, m)	28.24	1.64 (1H, m)	27.15
**12b**	2.16 (1H, m)		2.16 (1H, m)		2.16 (1H, m)	
**13**	1.87 (1H, m)	38.74	1.81 (1H, m)	39.75	1.88 (1H, m)	38.88
**14**		60.26		60.26		60.16
**15a**	1.16 (1H, m)	23.51	1.16 (1H, m)	24.94	1.16 (1H, m)	23.53
**15b**	1.71 (1H, m)		1.71 (1H, m)		1.71 (1H, m)	
**16a**	1.61 (1H, m)	37.27	1.59 (1H, m)	37.48	1.61 (1H, m)	37.2
**16b**	1.69 (1H, m)		1.68 (1H, m)		1.69 (1H, m)	
**17**		42.37		42.66		42.31
**18**	1.56 (1H, m)	50.71	1.38 (1H, m)	51.38	1.57 (1H, m)	50.57
**19**	2.77 (1H, m)	49.31	2.77 (1H, m)	48.95	2.76 (1H, m)	50
**20**	2.42 (1H, m)	37.17	2.39 (1H, m)	37.09	2.43 (1H, m)	36.62
**21a**	1.58 (1H, m)	25.07	1.58 (1H, m)	25.21	1.58 (1H, m)	25.07
**21b**	1.72 (1H, m)		1.72 (1H, m)		1.72 (1H, m)	
**22a**	0.99 (1H, s)	40.33	0.99 (1H, s)	40.74	0.99 (1H, s)	40.35
**22b**	1.41 (1H, s)		1.41 (1H, s)		1.44 (1H, s)	
**23**	0.93 (3H, s)	28.31	0.93 (3H, s)	28.27	0.91 (3H, s)	27.71
**24**	0.83 (3H, s)	22.18	0.83 (3H, s)	22.18	0.84 (3H, s)	22.85
**25**	0.89 (3H, s)	16.54	0.90 (3H, s)	16.53	0.89 (3H, s)	16.53
**26**	1.13 (3H, s)	17.21	1.13 (3H, s)	17.24	1.15 (3H, s)	17.13
**27**		179.5		179.11		177.44
**28**	0.82 (3H, s)	18.42	0.79 (3H, s)	18.15	0.81 (3H, s)	18.42
**29**	9.63 (1H, s)	205.39	9.87 (1H, s)	207.52	9.87 (1H, s)	205.13
**30**	1.03 (3H, d, 7.29)	7.3	1.05 (3H, d, 7.29)	14.17	1.04 (3H, d, 6.84)	7.25
**31**						170.75
**32**					2.05 (3H, s)	20.39

3*α*-hydroxy-19*α*-hydrogen-29-aldehyde-27-lupanoic acid (**2**): colorless acicular crystals, mp 200–201°C, purity 99.10%. [α]20 D = -9.48 (c = 0.29, MeOH). IR (KBr, max, cm^-1^): 3638, 2945, 2858, 1710, 1230, 1213. ^1^H and ^13^C NMR (CHCl_3_) (see Table 1). HR-ESI-MS: 495.3517 [M + Na]^+^) ([C_30_H_48_O_4_+ Na]^+^, calc: 495.3553).

3*α*-acetyl-19*α*-hydrogen-29-aldehyde-27-lupanoic acid (**3**): white amorphous powder, mp 200–201°C, purity 96.35%. [*α*]20 D = -9.06 (c = 0.29, MeOH). IR (KBr, max, cm^-1^): 3635, 2943, 2865, 1718, 1234, 1210. ^1^H and ^13^C NMR (CHCl_3_) (see Table 1). HR-ESI-MS: 537.3481 [M + Na]^+^) ([C_32_H_50_O_5_+ Na]^+^, calc: 537.3658).

### *In vitro* cell cytotoxicity assay

The cytotoxic effects of Compounds **1**–**3** were estimated *in vitro* against HepG-2, MCF-7 and T-84 tumor cell lines by Cell Counting Kit-8 assay [28]. Briefly, the cells were seeded into 96-well plates in triplicate at an initial number of 5,000 cells per well and cultured at 37°C, 5% CO_2_ for 24h, then treated with 0, 15, 20, 25, 30 μM of samples for another 24h. Ten microliters of the kit reagent was added to each well, and 2 h after all plates were scanned by a microplate reader (Thermo Fisher Scientific) at 450 nm. Cell cytotoxicity was calculated on the basis of absorbency.

### Apoptosis analysis

The method for apoptosis analysis referred to the literature [29]. Apoptosis was discriminated with the annexin V-FITC/propidium iodide test. Cells were seeded at 3 × 10^5^/well in 10% FBS-DMEM into 6-well plates, and treated with Compound **1** with various concentrations. The cells were washed twice with cold Phosphate Buffered Saline (PBS) and then resuspended in 1 × Binding Buffer (0.1 M Hepes/NaOH (pH 7.4), 1.4 M NaCl, 25 mM CaCl_2_) at a concentration of 1 × 10^6^ cells/mL. 100 μL of the solution (1 × 10^5^ cells) was transferred to a 5 mL culture tube, and 5 μL of FITC Annexin V (BD, Pharmingen) and 5 μL of propidium iodide (PI) were added to each tube. The cells were gently vortexed and incubated at 25°C for 15 min in the dark. Then 200 μL of PBS was added to each tube. Analysis was performed with the system software (Cell Quest; BD Biosciences). The percentage of cells positive for PI and/or Annexin V-FITC was reported inside the quadrants. Lower left quadrant, viable cells (annexin V-/PI-); lower right quadrant, early apoptotic cells (annexin V+/PI-); upper right quadrant, late apoptotic cells (annexin V+/PI+); upper left quadrant, necrotic cells (annexin V-/PI+).

### Hoechst 333258 staining

HepG-2 cells grown on a sterile cover slip in 6-well plates were treated with Compound **1** for 24 h. The culture medium containing Compound was removed, and the cells were fixed in 4% paraformaldehyde for 10 min. After two PBS washes, the cells were stained with 0.5 mL of Hoechst 33258 (Beyotime, Haimen, China) for 5 min and again two PBS washes. The stained nuclei were viewed using a Nikon ECLIPSETE2000-S fluorescence microscope (OLYMPUS Co., Japan) under 350 nm excitation and 460 nm emissions.

### AO/EB staining

HepG-2 cells were seeded on a sterile cover slip in 6-well tissue culture plates at a concentration of 5 ×10^4^ cell/mL in a volume of 2 mL. Following incubation, the medium was removed and replaced with fresh medium plus 10% fetal bovine serum and supplemented with concentrations of Compound **1** for 24 h. After the treatment period, the cover slip with monolayer cells was inverted on a grass slide with 10 μL of AO/EB stain (100 mg/mL). Fluorescence was read on a Nikon ECLIPSETE2000-S fluorescence microscope.

### ROS assay

HepG-2 cells were seeded into 6-well plates for 24 h and subjected to various treatments. Then, the cells were cultured in a cell-free medium solution containing 10 mM DCFH-DA (Beyotime, Haimen, China) at 37°C for 30 min in dark, and then with 3 times PBS washes. Cellular fluorescence was quantified under Nikon ECLIPSETE2000-S fluorescence microscope at 485 nm excitation and 538 nm emission.

### Mitochondrial membrane potential staining

Mitochondrial depolarization was surveyed using cationic lipophilic dye JC-1 (Beyotime, Haimen, China) in MGC-803 cells. Briefly, cells were cultured with an equal volume of JC-1 staining solution (3 mg/mL) at 37°C for 20 min after incubated in 6-well plates and subjected to indicated treatments, and then washed twice with PBS. The changes in mitochondrial membrane potentials were measured by determining the relative amount of dual emissions from mitochondrial JC-1 monomers or aggregates using flow cytometry. Mitochondrial depolarization was identified by green/red fluorescence intensity ratio.

### Western blot

HepG-2 cells were collected after treatments with Compound **1** (0, 15 and 30 μM) for 24 h and then lysed in ice-cold RIPA buffer (1×PBS, 1% NP-40, 0.5% sodium deoxycholate and 0.1% SDS) which containing 100 μg/mL PMSF, 5 μg/mL Aprotinin, 5 μg/mL Leupeptin, 5 μg/mL Pepstatin and 100 μg/mL NaF. After centrifugation at 12,000 rpm for 10 min, the protein in the supernatant was quantified by the Bradford method using Multimode varioscan instrument (Thermo Fischer Scientifics). 30 μg protein per lane was applied in 12% SDS polyacrylamide gel. After electrophoresis, the protein was transferred to a polyvinylidine difluoride (PVDF) membrane (Amersham Biosciences). The membrane was blocked in TBST containing 5% blocking powder (Santacruz) at room temperature for 2 h. The membrane was washed with TBST for 5 min, and primary antibody (Bax, Bcl-2, cytochrome c, caspase-9, -3) was added and incubated at 4°C overnight (O/N). After three washes in TBST, the membrane was cultivated with corresponding horseradish peroxidase-labeled secondary antibody (1:20000) (Santa Cruz) at room temperature for 1 h. Membranes were rinsed with TBST for three times, 15 min each, and the protein blots were visualized with chemiluminescence reagent (Thermo Fischer Scientifics Ltd.). The X-ray films were developed with developer and fixed with fixer solution.

### Statistics

The data were processed by the Student’s *t*-test with the significance level p < 0.05 and 0.01 using SPSS.

## Results and discussion

### Chemistry structures

Compound **1** was obtained as a white amorphous powder, [*α*]20 D = -10.00 (c = 0.29, MeOH). Its molecular formula was established as C_30_H_48_O_4_ by HR-ESI-MS which showed an [M + Na]^+^ ion peak at *m*/*z* 495.3558 (calculated for [C_30_H_48_O_4_+ Na]^+^: 495.3553), indicating 7 degrees of unsaturation.

There are six methyl group signals at *δ* 0.82, 0.83, 0.89, 0.93, 1.04, 1.13 in the ^1^H NMR spectrum and 30 carbon signals in ^13^C NMR spectrum, which suggested that compound **1** was a triterpenoid [30]. *δ*_H_ 1.04 (d, 3H, *J* = 7.29) indicated that this methyl group coupled with a methine proton. The carbon signal at *δ* 205.39 and the proton signal at *δ* 9.63 exhibited one aldehyde group. Besides, a carboxyl group (*δ*c 179.50) was clearly observed in NMR spectra data. The proton and carbon signals were shown in Table 1.

**Fig 2:**
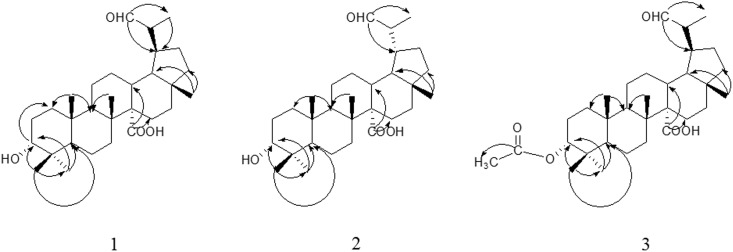
Structures and Key HMBC correlations (C→H) of Compound 1–3.

A series of 2D NMR spectra were employed to further determine the structure of Compound **1**. H-19 (*δ* 2.77) correlated with C-19 (*δ* 49.95) in HSQC spectrum indicated that this compound was a lupane-triterpenoid [21]. Then, the carbon signal C-29 (*δ* 205.39) crossed with H-30 at *δ* 1.04 (3H, d, *J* = 7.29) and H-19 (*δ* 2.77); the correlations between C-30 (*δ* 7.30) and H-19 proved aldehyde group located at C-29 by HMBC spectrum. Next, it was confirmed that a carboxylic group located at C-14 rather than C-17 through the following evidences: the carboxyl carbon signal (*δ* 179.50) showed cross with H-13 (*δ* 1.87) and H-15 (*δ* 1.16 and *δ* 1.71); the quaternary carbon C-14 (*δ* 60.26) correlated with the protons at *δ* 1.13 (3H-26), *δ* 1.87 (H-13) and *δ* 1.56 (H-18); the methyl signal of C-28 (*δ* 18.42) exhibited cross peaks with H-18 (*δ* 1.56) and H-22 (*δ* 1.41). After that, an oxygen-bearing methine proton at *δ* 3.42 (t, 1H) correlated with the carbon signal at δ 76.19 confirmed that a hydroxyl was attached to the structure (HSQC), what’s more, the hydroxyl was located at C-3 according the HMBC cross-peaks of the proton *δ* 3.42 with C-23 (*δ* 28.31) and C-24 (*δ* 22.18). Key HMBC correlations were shown in Fig 2. Thus, the planar structure of **1** was determined as 3-hydroxy-29-aldehyde-27-lupanoic acid.

**Fig 3:**
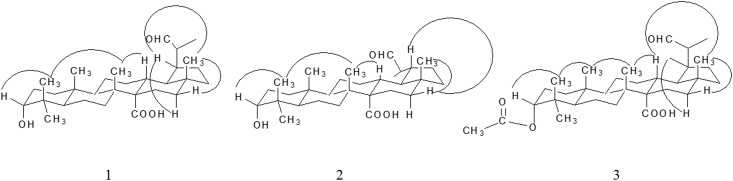
Key ROESY correlations of Compound 1–3.

The relative configuration of **1** was determined by a ROESY experiment. All chiral centers were assumed to be consistent with (20s)-3*α*, 29-dihydroxylupan-27-oic acid [21]. The ROESY correlation of H-3/H_3_-24/H_3_-26/H-13/H*β*-16/H_3_-28 indicated these protons were *β*-orientation. In addition, the cross of H-19/H*α*-16 proved H-19 was α-equatorial and the isopropylaldehyde group located at C-19 was *β*-orientation (Fig 3). On the basis of the above results, the structure **1** was deduced as 3*α*-hydroxy-19*α*-hydrogen-29-aldehyde-27-lupanoic acid.

Compound **2** was isolated as a colorless acicular crystal, [α]20 D = -9.48 (c = 0.29, MeOH). Its molecular formula was assigned to be C_30_H_48_O_4_ on the basis of the HR-ESI-MS, which gave an [M + Na]^+^ ion peak at *m*/*z* 495.3517 (calculated for [C_30_H_48_O_4_+ Na]^+^: 495.3553).

Similar to compound **1**, the NMR spectra data of **2** showed the characteristic signals of six methyl groups, an aldehyde group, a carboxyl group and a hydroxyl group. And the planar structure of **2** had been proved by HSQC and HMBC spectra, which was the same as the previous one (Table 1; Fig 2). However, its carbon chemical shifts of C-29 (*δ* 14.17) and C-30 (*δ* 207.52) were quite different with that of compound **1** (C-29 at *δ* 7.30 and C-30 at *δ* 205.39). Furthermore, the ROESY cross of H-19/H*β*-16 proved that the isopropylaldehyde group at C-19 was *α*-orientation, which was different from Compound **1** (H19/H*α*-16). Hence, the structure **2** was identified as 3*α*-hydroxy-19*β*-hydrogen-29-aldehyde-27-lupanoic acid.

Compound **3** was obtained as a white amorphous powder with [*α*]20 D = -9.06 (c = 0.29, MeOH). Its molecular formula, C_32_H_50_O_5_, was established by ^13^C NMR and HR-ESI-MS (*m*/*z* 537.3481 [M + Na]^+^, calculated for [C_32_H_50_O_5_ + Na]^+^, 537.3658) data, corresponding to eight indices of hydrogen deficiency.

The assigned NMR data of **3** were in Table 1. A proton signal at *δ* 9.64 together with the carbon signal at *δ* 205.13 helped to testify the aldehyde group, which was quite similar with Structure **1**. However, in Compound **3**, seven methyl group signals were clearly observed in the ^1^H-NMR spectra, besides, two carbonyl groups were found based on the carbon signals at *δ* 170.75 and *δ* 177.44. In HMBC, the signal (*δ*_H_ 2.05 (s, 3H) correlated with *δ*_C_ 170.75) indicated that an acetyl group was attached to the structure (Fig 2). Moreover, the fact of a methine proton at *δ* 4.61 (t, 1H) correlated with C-23 (*δ* 27.71) and C-24 (*δ* 22.85) confirmed that the acetyl was located at C-3 (HMBC), which substituted the hydroxyl group of Compound **1**. The ROESY correlation of H-3/H_3_-24/H_3_-25/H_3_-26/H-13/H*β*-16/H_3_-28 indicated these protons were *β*-orientation; the cross of H-19/H*α*-16 proved H-19 was α-equatorial, which was similar to Compound **1** (Fig 3). Based on these results, structure **3** was assigned as 3*α*-acetyl-19*α*-hydrogen-29-aldehyde-27-lupanoic acid.

Compounds **4**–**9** were identified by comparing their spectrum data with literature values as follows: tormentic acid (**4**) [31]; ursolic acid (**5**) [32]; Betulinic acid (**6**) [33]; 3, 3’, 4’-tri-O-methylellagic acid (**7**) [34]; Luteolin (**8**) [35]; Quercetin (**9**) [36].

### Evaluation of antitumor activities

Quite a number of triterpenoids with different structures have been confirmed to be antitumor agents. In the past few years, plenty of literatures about pentacyclic triterpenoids with potent anti-cancer activities were published [37]. It is well-known that the inhibition on cancer proliferation has been a continuous effort in cancer treatment. Therefore, *in vitro* cytotoxicity of Compounds **1**–**3** were evaluated by CCK-8 assay against HepG-2, MCF-7 and T-84 cell lines, with matrine as a positive control as shown in Table 2. Compound **1** demonstrated strong activities against HepG-2 cell line with IC_50_ value of 17.84 μM, comparable to the positive control (matrine) with IC_50_ value of 30.88 μM, while it exhibited moderate cytotoxic activities for the other two cell lines with IC_50_ values 30.78 (MCF-7) and 27.89 (T-84) μM, respectively. Compound **2** showed similar activities to Compound **1**, with IC_50_ values of 18.21, 30.99 and 27.87 μM, respectively; Compound **3** exhibited relatively weaker cytotoxicity for MCF-7 (IC_50_ 35.43 μM), and the lowest cytotoxicity for T-84 (IC_50_ 40.64 μM).

**Table 2:** Cytotoxicity (IC_50_) of Compounds 1, 2 and 3 (n = 3).

Sample	Cytotoxicity (IC_50_, μM)		
	HepG-2	MCF-7	T-84
**1**	17.84±1.21**	30.78±1.88*	27.89±1.96*
**2**	18.21±1.94**	30.99±1.83*	27.87±0.99*
**3**	25.32±1.87*	35.43±1.52	40.64±3.64**
**Matrine**^a^	30.88±2.28	35.54±2.92	32.81±2.95

^a^ Positive control;

* and**, Significant difference between Compound and Matrine by *t*-test at p < 0.05 and 0.01, respectively.

With the investigation, we found that Compound **1** and **2** exhibited pretty similar activities against all these three cell lines (Table 2, S1 Table), suggesting that the change of isopropyl aldehyde configuration has little effect on the antitumor activity. Meanwhile, Structure **1**–**3** all displayed higher resistance to HepG-2 cell line which indicate that specific differences in chemical structure with a carboxyl group at C-14 have an influence on the cytotoxic properties and proliferation of HepG-2 cell line (Table 2, *p* < 0.05 and 0.01). What is interesting is that this result was similar to the previously reported by Sun *et al*. [38], who discovered that an oleanane-type triterpene with a carboxyl group at C-14 from *Astilbe chinensis* could significantly inhibit on the proliferation of HeLa cells and induce cell apoptosis. In particular, although Compound **3** also showed activity against HepG-2, its inhibition level was much lower than that of Compound **1** (S1 Table, *p* < 0.01). In view of these differences of the two structures, it can be concluded that hydroxyl group could be more important for the enhanced cytotoxic activity than acetyl.

**Fig 4:**
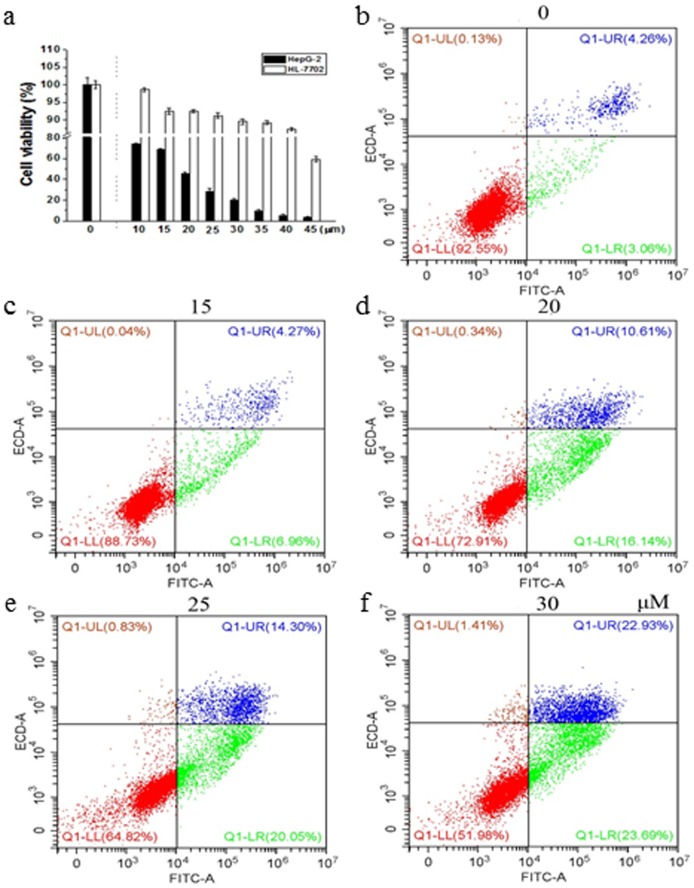
(a) Effects of Compound 1 on human hepatocellular carcinoma cell line (HepG-2) and normal liver cell line (HL-7702). (b-f) Apoptosis analysis of HepG-2 cells treated with compound 1 at various concentrations of 0, 15, 20, 25, 30 μM, respectively.

Due to its well cytotoxic inhibition on HepG-2, the inhibition activities of Compound **1** against HL-7702 normal human liver cell line were also assayed (Fig 4a). With respect to cell viability, above 90% HL-7702 cells were survival even when the concentration of Compound **1** was 35 μM. In contrast, with the improvement of sample concentration, the cell viability of HepG-2 gradually decreased, with only a survival rate of 9.50% when treated with Compound **1** at a concentration of 35 μM. These results indicated that **1** showed low cytotoxicity on normal human liver cell line HL-7702, on the contrary high *in vitro* antiproliferative activity on the cancer cell lines, which indicated that the targeted compounds had selective and significant effect on the HepG-2 cell lines.

### Effects of Compound 1 on the induction of apoptosis

A reduction in cell growth and induction in cell death are two major means to inhibit tumor growth [39]. Apoptosis is a complex physiological process that permits the reduction of harmful or unnecessary cells during development, tissue homeostasis and disease. To confirm whether Compound **1** induced reduction in cell viability due to the induction of apoptosis, the apoptotic rates of HepG-2 cells treated with Compound **1** at various concentrations of 0, 15, 20, 25, 30 μM for 24 h were determined in present work (Fig 4b–4f). It was observed that Compound **1** significantly caused cell apoptosis at both early and late stages. Specifically, compared with control (3.06%, 4.26%), the early and late apoptosis rates were gradually increased from (6.96%, 4.27%), (16.14%, 10.61%) to (20.05%, 14.30%) and (23.69%, 22.93%) after treatment at 15, 20, 25, 30 μM for 24h, respectively. These results provided evidence that Compound **1** could cause a notable increase of cellular apoptosis in a dose-dependent manner from 0 to 30 μM.

### Hoechst 33258 and AO/EB staining

**Fig 5:**
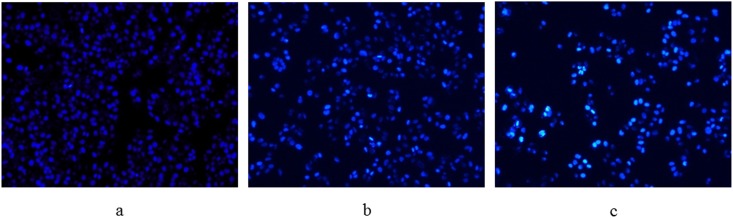
Hoechst 33258 staining of HepG-2 cells treated with Compound 1. (a) HepG-2 cells without Compound 1 treatment were used as control and (b, c) HepG-2 cells treated with Compound 1 (15 μM and 30 μM) for 24 h, respectively.

To further validate cell apoptosis upon treatment of Compound **1**, HepG-2 cells were stained with Hoechst 33258 and AO/EB staining after the treatment for 24 h at different concentrations (0, 15 and 30 μM). Hoechst 33258 is a membrane permeable blue fluorescent dye which stains the cell nucleus. Under fluorescence microscope, live cells exhibited uniformly light blue nuclei after treatment with Hoechst 33258, while apoptotic cells had bright blue nuclei because of karyopyknosis and chromatin condensation, besides dead cells’ nuclei could not be stained. Our observation showed that most of the normal cells exhibited weak blue fluorescence in the control group (Fig 5). While in the treatment group with **1**, apoptotic cells increased gradually in a dose-dependent manner and exhibited typical changes including reduction of cellular volume, bright staining and condensed or fragmented nuclei.

**Fig 6:**
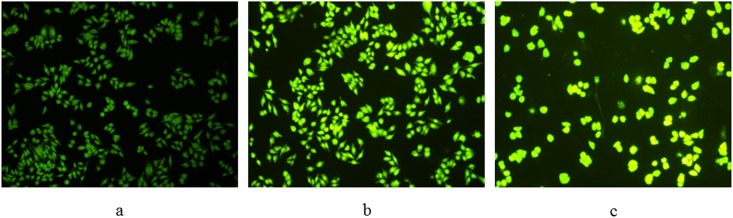
AO/EB staining of HepG-2 cells treated with Compound 1. (a) Not treated with Compound 1 were used as control and (b, c) treatment with Compound 1 (15 μM and 30 μM) for 24 h, respectively.

Apoptosis was further evaluated using acridine orange/ethidium bromide (AO/EB) double staining, which differentiates between necrosis and apoptosis by the difference in membrane integrity. AO is a vital dye which can pass through cell membranes of living or early apoptotic cells, while EB can only stains cells that had lost their membrane integrity. In our research, the cytotoxicity of Compound **1** at 15 and 30 μM for 24 h treatment against HepG-2 cells was detected by AO/EB staining, and cellstreated without **1** were used as control. As shown in Fig 6, cells treated with compound at different concentrations had obviously changed. The nuclei were stained as yellow green, the morphology showed pycnosis. These findings also confirmed that Compound **1** was able to induce apoptosis, which consistent with the results of Hoechst 33258 staining.

### Intracellular ROS level in HepG-2 cells induced by Compound 1

**Fig 7:**
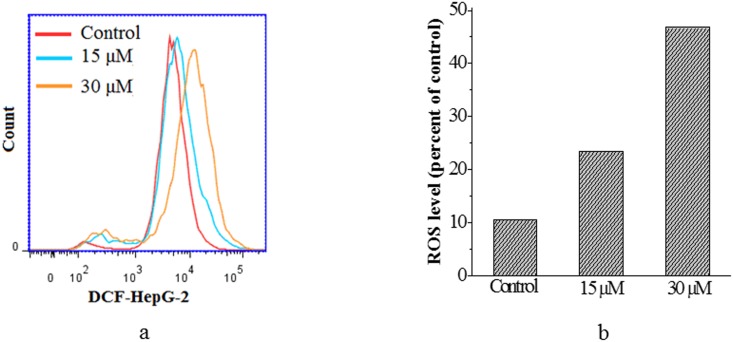
Compound 1 increased the intracellular ROS level of HepG-2 cells. (a) Increase of ROS level in HepG-2 cells showing a concentration dependent manner. (b) ROS levels in HepG-2 cells expressed as units of MFI were calculated as a percentage of the control.

Reactive oxygen species (ROS) are highly harmful elements to cells as they initiate oxidative stress and ultimately cause cellular damage. Excessive ROS generation renders cells vulnerable to apoptosis [40, 41]. Several studies have shown that natural pentacyclic triterpenes trigger a rapid production of intracellular ROS, which might be responsible for their cytotoxic actions [42, 43]. To determine whether **1** triggers ROS generation in HepG-2 cells to induce apoptosis, the ROS level in the cells with or without **1** treatment was measured using 2,7-dichlorofluorescein diacetate (DCF-DA) as fluorescent probe by flow cytometry. As shown in Fig 7, the results showed that Compound **1** induced an increase of ROS level in HepG-2 cells. After exposure to 15 μM Compound **1** for 24 h, the ROS level increased to 23.5%, more than two times higher than that of control (10.6%). Even further ROS level increased to 46.8% when treated with **1** at the concentration of 30 μM for 24 h. Taken together, these results indicate that Compound **1** can cause the oxidative imbalance in HepG-2 cells. This induction of oxidative burst is a key factor behind the anti-proliferative activity of Compound **1**.

### Mitochondrial membrane potential (Δ*Ψ*m) in HepG-2 cells

**Fig 8:**
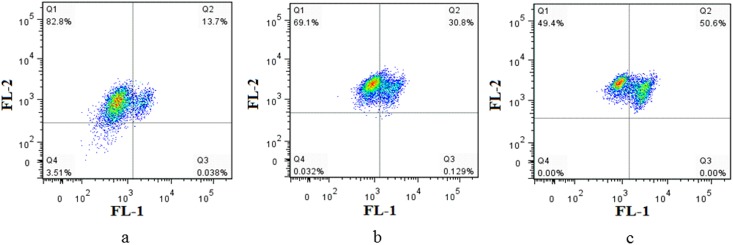
Compound 1 decreased the ΔΨm level of HepG-2 cells. (a, b, c) Decrease of the ΔΨm level in HepG-2 cells showing a dose-dependent manner.

The loss of Δ*Ψ*m is regarded as a limiting factor in the apoptotic pathway. To further investigate the apoptosis-inducing effect of target compound, changes of mitochondrial membrane potential were detected using the fluorescent probe JC-1, which can easily pass through the plasma membrane into cells and accumulates in mitochondria [44]. As indicated in Fig 8, the treatment of HepG-2 cells with Compound **1** at different concentrations for 24h led to the loss of Δ*Ψ*m. After exposed to 15 and 30 μM target compound for 24 h, Δ*Ψ*m was reduced to 69.1% and 49.4% of control, respectively, in a dose-dependent manner. The experimental results demonstrated that depolarization of mitochondria were occurred after treated with Compound **1**.

### Release of cytochrome c and activation of caspases were involved in the apoptosis induced by Compound 1

**Fig 9:**
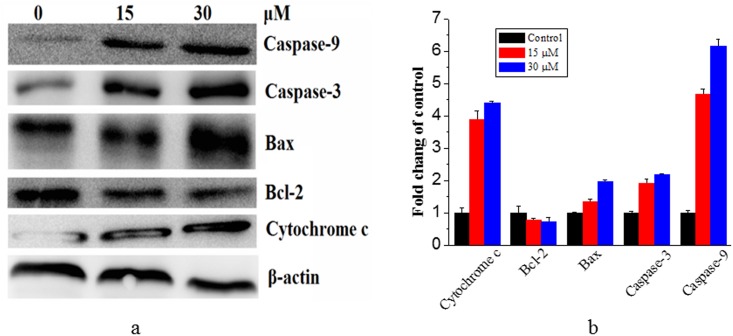
Compound 1 treatment led to the activation of cytochrome c, Bax, Bcl-2, caspase-9 and caspase-3. Densitometric analysis of western blot results of panel (a), and the results are shown in (b). The results were expressed as the mean ± SEM of three experiments (p< 0.05).

Mitochondrial pathway, which is connected with the loss of Δ*Ψ*m in most of times, is one of the major ways to induce cellular apoptosis. Because the loss of Δ*Ψ*m has been suggested to cause the release of cytochrome c from the mitochondria to the cytosol, which is a limiting factor in the mitochondrial pathway. To confirm the molecular mechanisms involved in the observed apoptosis, we investigated the effects of Compound **1** on the expression of proteins related with mitochondria mediated apoptosis. In Fig 9a, the cytochrome c level in the cytosol was gradually improved when treated with Compound **1** at concentrations of 0, 15 and 30 μM, most probably due to the release of mitochondrial cytochrome c. At the same time, it is well-known that mitochondrial apoptotic pathway is regulated by the Bcl-2 family of pro- and anti-apoptotic proteins, which stimulate the permeabilization of the mitochondrial outer membrane and cytochrome c released into the cytosol, promoting in the activation of the caspase cascade and induction of apoptotic cell death [45, 46]. As shown in Fig 9, compared with the control group, **1** induced a significant increase of Bax level and an inhibition on the expression of Bcl-2, in a dose-dependent manner. Cytochrome c is reportedly involved in the activation of downstream caspases that trigger apoptosis, so these results indicated that caspases are involved in the apoptotic process downstream of mitochondria. In this study, we examined the roles of important caspases (caspase-9 and -3) in the cellular response to Compound **1.** Western blot analysis showed that treatment of HepG-2 cells with **1** significantly induced cleavage of caspase-9 and -3. These results revealed that caspases are involved in the intrinsic apoptotic process downstream of mitochondria. From the above, Compound **1** induced HepG-2 cells apoptosis possibly by decreasing the activation of Bcl-2 and stimulating its downstream proteins associated with mitochondria-dependent apoptotic pathway.

Plants used in traditional medicine, ethnomedicine, folk medicine and herbalism provide a rational and obvious source of candidates for the targeted identification of lead substances. In this study, all nine compounds were classified into three categories: triterpenes (**1**–**6**), phenols (**7**) and flavonoids (**8**, **9**). Three new compounds were found rarely, although six known structures occur widely in majority of the botanical taxa. Combined with other studies, some ellagic acid derivatives are considered to be useful taxonomic markers for this genus and triterpenoids are the main substances isolated from the *Potentilla* species[15, 16]. For this reason, several more recent phytochemical studies have concentrated on the isolation of triterpenoid structures from *Potentilla* species, comprising *Potentilla erecta*, *Potentilla anserine*, *Potentilla multicaulis* and *Potentilla discolor*. These compounds are usually based on pentacyclic triterpene skeleton [16], which represent a very powerful class of natural products due to their broad biological activity and amazing diversity of structures [25]. It is well-known that lupane-triterpenoids are considered as a particularly important series of pentacyclic triterpenoids, and they abundantly occur in the plant kingdom and other organisms. Whereas, to the best of our knowledge, the lupane-triterpenoids showing the presence of a carboxyl group at C-14 position is present in a limited number of natural resources. So far, sporadic C-27-carboxylated-pentacyclic triterpenoids have been obtained from saxifragaceae plants [27, 38, 47]. In fact, as mentioned before, Yang *et al*. [21] isolated two new lupane-triterpenoids from the whole herbs of PD with their C-27 position were highly oxygenated. Combined with the previous work, PD may be used as another important resource for these markable compounds.

Cancer has become one of leading cause of unnatural death globally. Over the ten decades, the development of antineoplastic drugs has greatly attracted scientists’ interest. In fact, at least 60% of anticancer agents originate from natural compounds [48]. Meanwhile, diabetes mellitus can lead to serious harm to human health like cancer [49]. Encouragingly, it was proved by Han *et al*. [47] that the pentacyclic triterpenoids substituted with a carboxylic acid at the C-27 position isolated from *Astilbe rivularis* can enhance glucose uptake, suggesting that C-27-carboxylated-pentacyclic triterpenoids may serve as scaffolds for development as agents for the management of blood glucose levels in disease states such as diabetes. Thus, all these observations confirmed that C-27-carboxylated-pentacyclic triterpenoids has a crucial effect on human health of two major killers.

## Conclusion

The present study revealed that three novel C-27-carboxylated-lupane-triterpenoids were isolated from *Potentilla discolor* Bunge, which were found rarely in nature. 3*α*-hydroxy-19*α*-hydrogen-29-aldehyde-27-lupanoic acid was confirmed that it had a selective and distinctive cytotoxicity toward HepG-2 cells, which supported the previous conclusion that the position of carboxyl group affects the cytotoxicity of pentacyclic triterpenes. Meanwhile, 3*α*-hydroxy-19*α*-hydrogen-29-aldehyde-27-lupanoic acid could cause a marked increase of HepG-2 cellular apoptosis in a dose-dependent manner. The further mechanisms of apoptosis demonstrated that this compound might decrease the activation of Bcl-2 and stimulate its downstream proteins which are associated with the mitochondria-dependent apoptotic pathway. All these results should be useful in the search for new potential antitumor agents and for developing semisynthetic lupane-triterpenoid derivatives with antitumor activities.

## Supporting information

S1 Fig ^1^H NMR spectrum of Compound 1. (TIF)

S2 Fig ^13^C NMR spectrum of Compound 1. (TIF)

S3 Fig HSQC spectrum of Compound 1. (TIF)

S4 Fig HMBC spectrum of Compound 1. (TIF)

S5 Fig ROESY spectrum of Compound 1. (TIF)

S6 Fig HR-ESI-MS of Compound 1. (TIF)

S7 Fig ^1^H NMR spectrum of Compound 2. (TIF)

S8 Fig ^13^C NMR spectrum of Compound 2. (TIF)

S9 Fig HSQC spectrum of Compound 2. (TIF)

S10 Fig HMBC spectrum of Compound 2. (TIF)

S11 Fig ROESY spectrum of Compound 2. (TIF)

S12 Fig HR-ESI-MS of Compound 2. (TIF)

S13 Fig ^1^H NMR spectrum of Compound 3. (TIF)

S14 Fig ^13^C NMR spectrum of Compound 3. (TIF)

S15 Fig HSQC spectrum of Compound 3. (TIF)

S16 Fig HMBC spectrum of Compound 3. (TIF)

S17 Fig ROESY spectrum of Compound 3. (TIF)

S18 Fig HR-ESI-MS of Compound 3. (TIF)

S19 Fig HPLC of Compound 1. (TIF)

S20 Fig HPLC of Compound 2. (TIF)

S21 Fig HPLC of Compound 3. (TIF)

S1 Table Cytotoxicity (IC_50_) comparison of Compounds 1, 2 and 3 at pair (n = 3). (TIF)
