# Supplementary figures and images for: Three new C-27-carboxylated-lupane-triterpenoid derivatives from Potentilla discolor Bunge and their in vitro antitumor activities
Source: PLoS One. 2017 Apr 7;12(4):e0175502. doi: 10.1371/journal.pone.0175502 (PMC5384777; doi:10.1371/journal.pone.0175502)

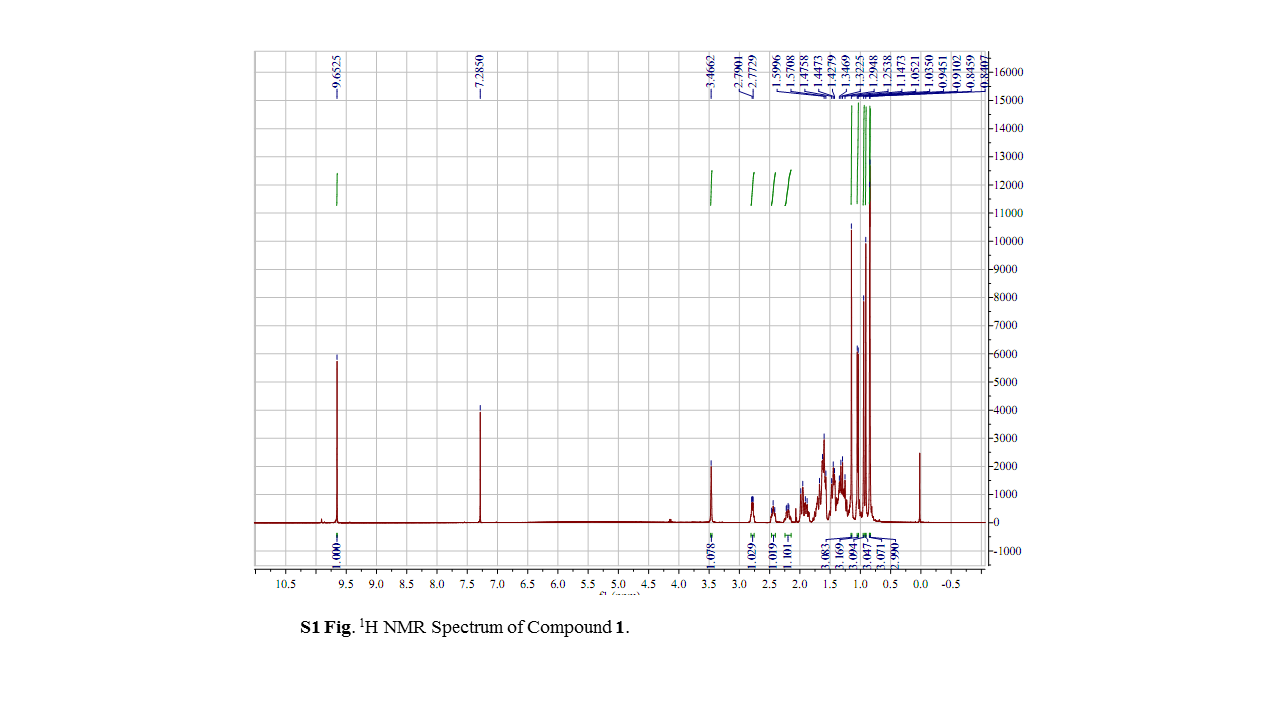

Supplement: S1 Fig — (TIF) [file pone.0175502.s001.TIF]

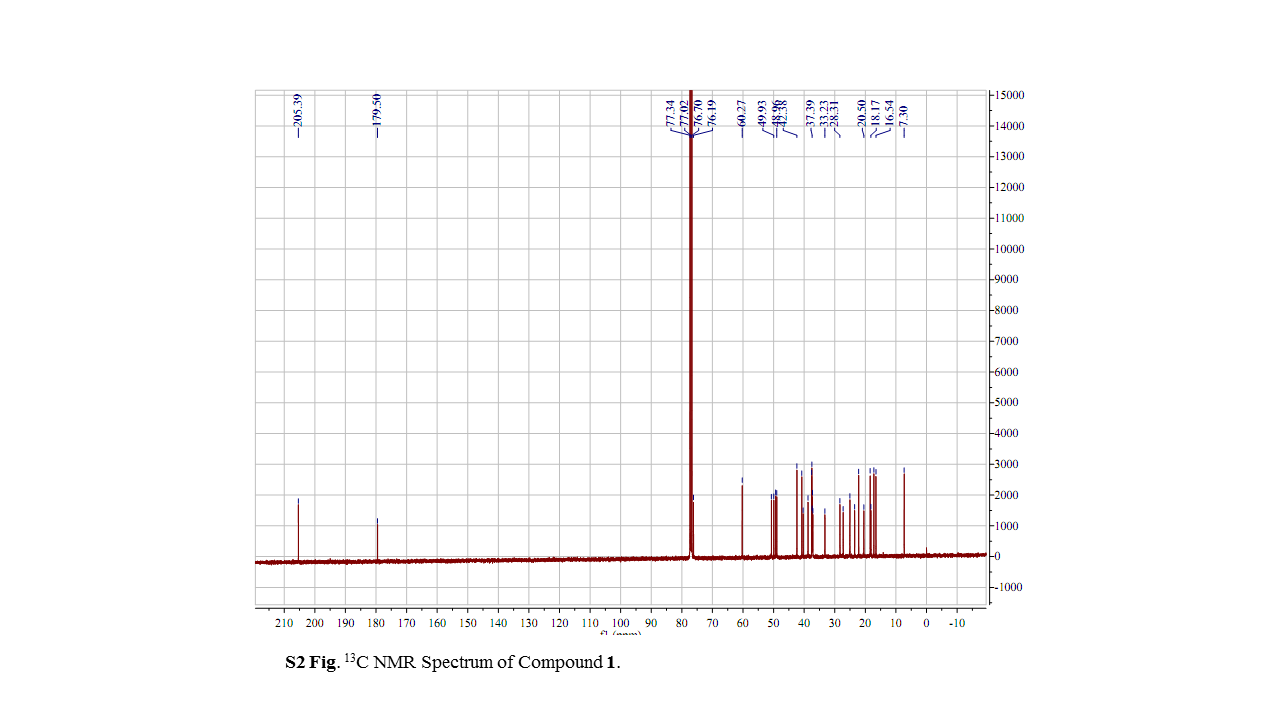

Supplement: S2 Fig — (TIF) [file pone.0175502.s002.TIF]

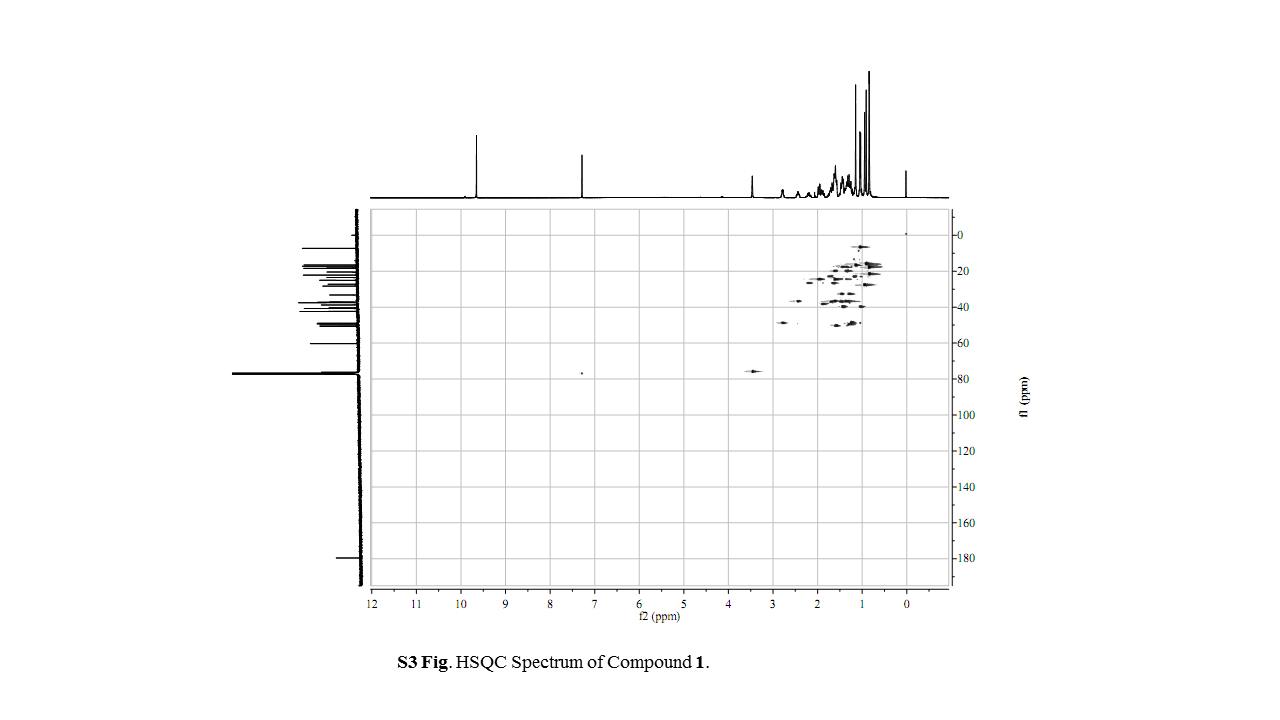

Supplement: S3 Fig — (TIF) [file pone.0175502.s003.TIF]

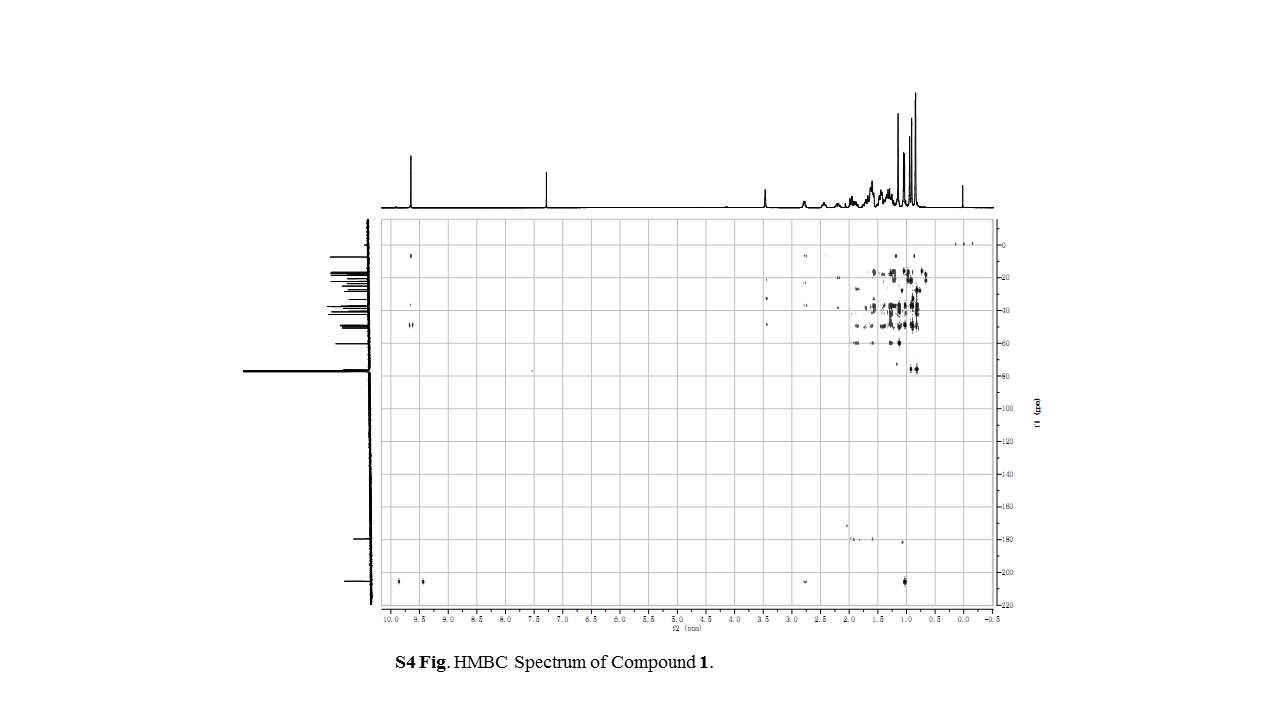

Supplement: S4 Fig — (TIF) [file pone.0175502.s004.TIF]

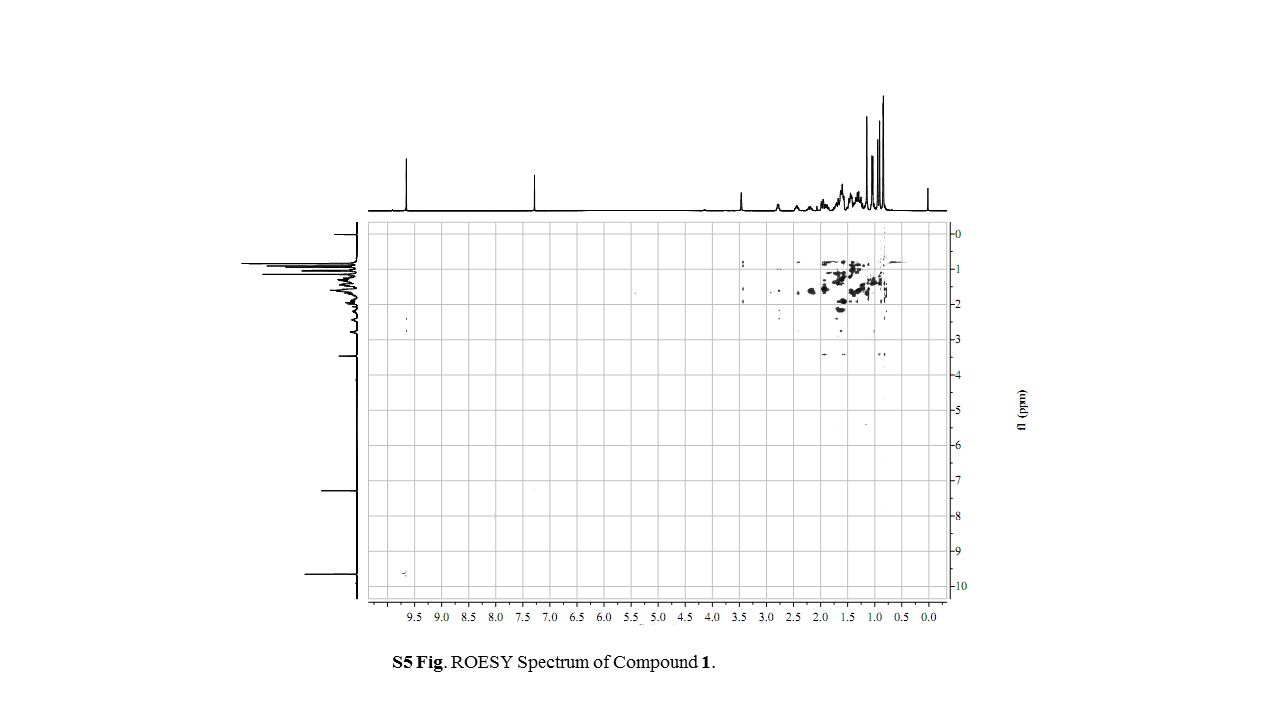

Supplement: S5 Fig — (TIF) [file pone.0175502.s005.TIF]

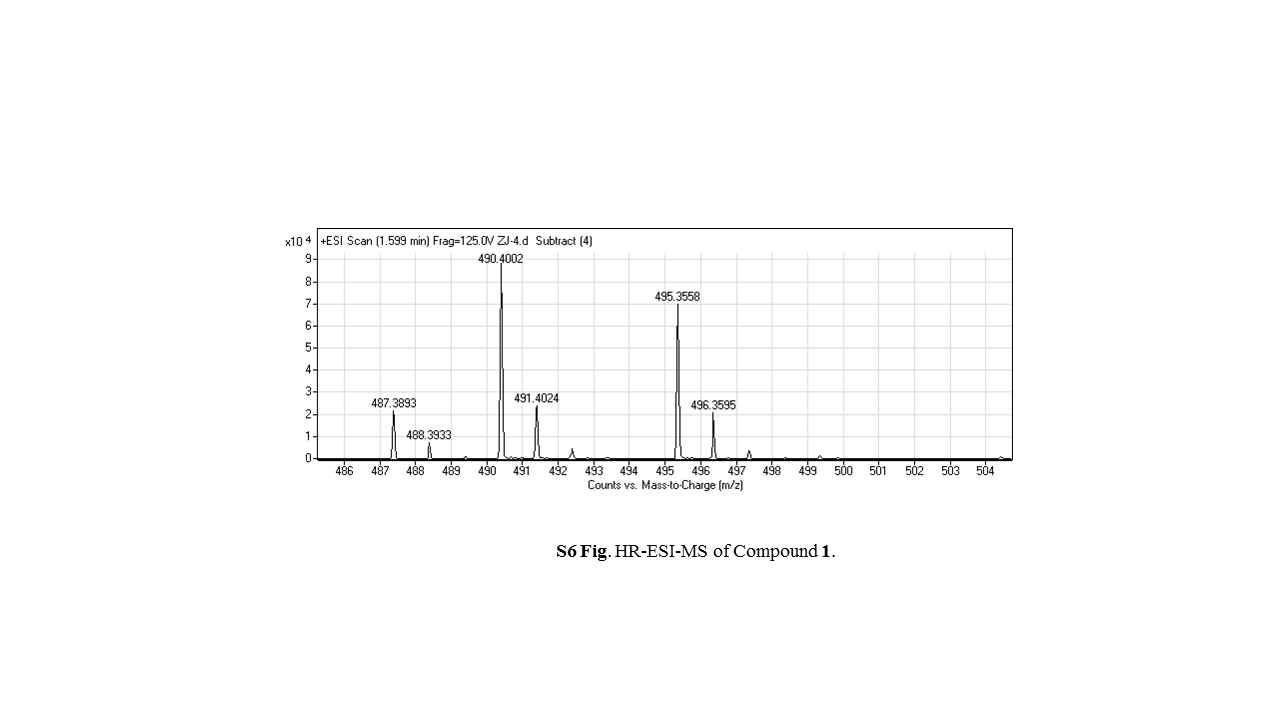

Supplement: S6 Fig — (TIF) [file pone.0175502.s006.TIF]

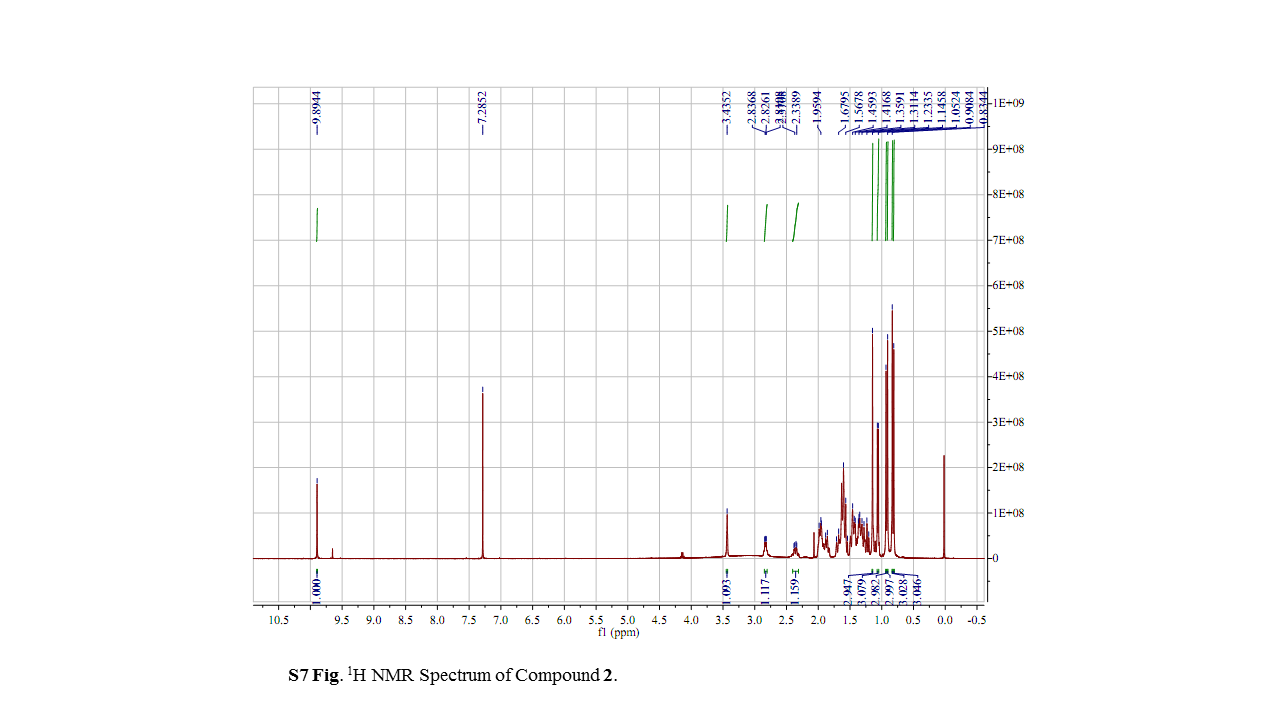

Supplement: S7 Fig — (TIF) [file pone.0175502.s007.TIF]

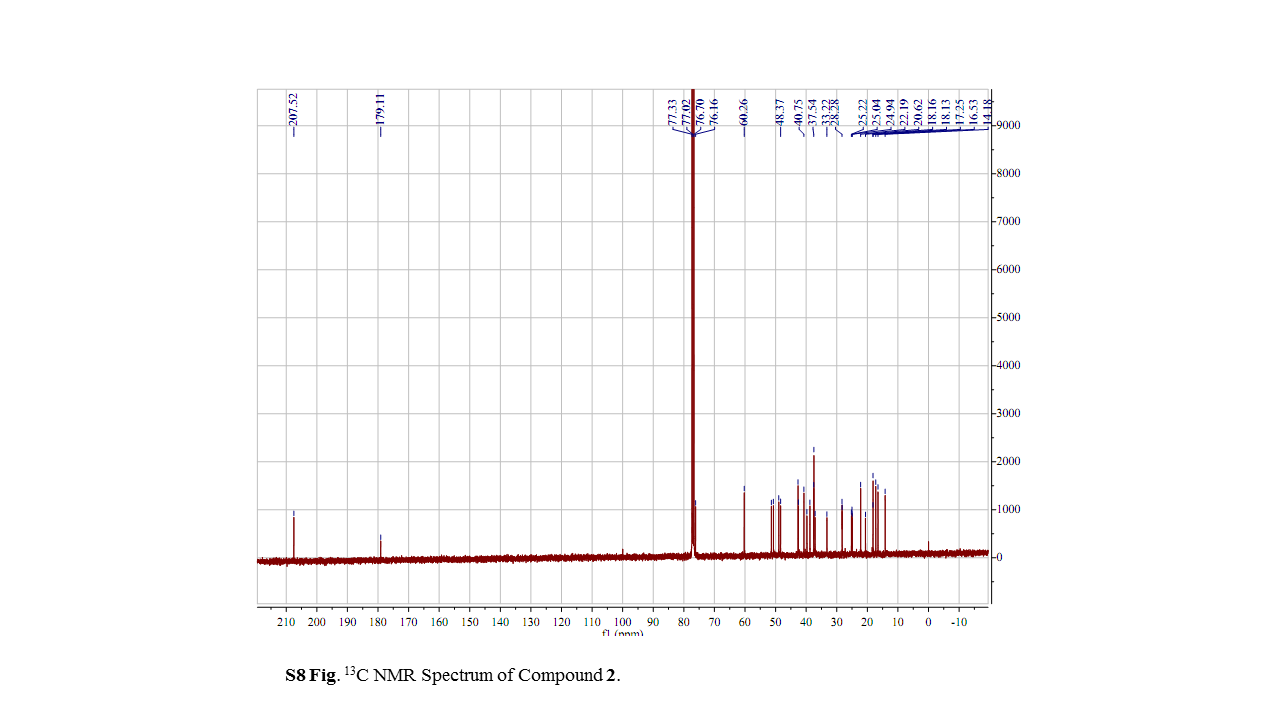

Supplement: S8 Fig — (TIF) [file pone.0175502.s008.TIF]

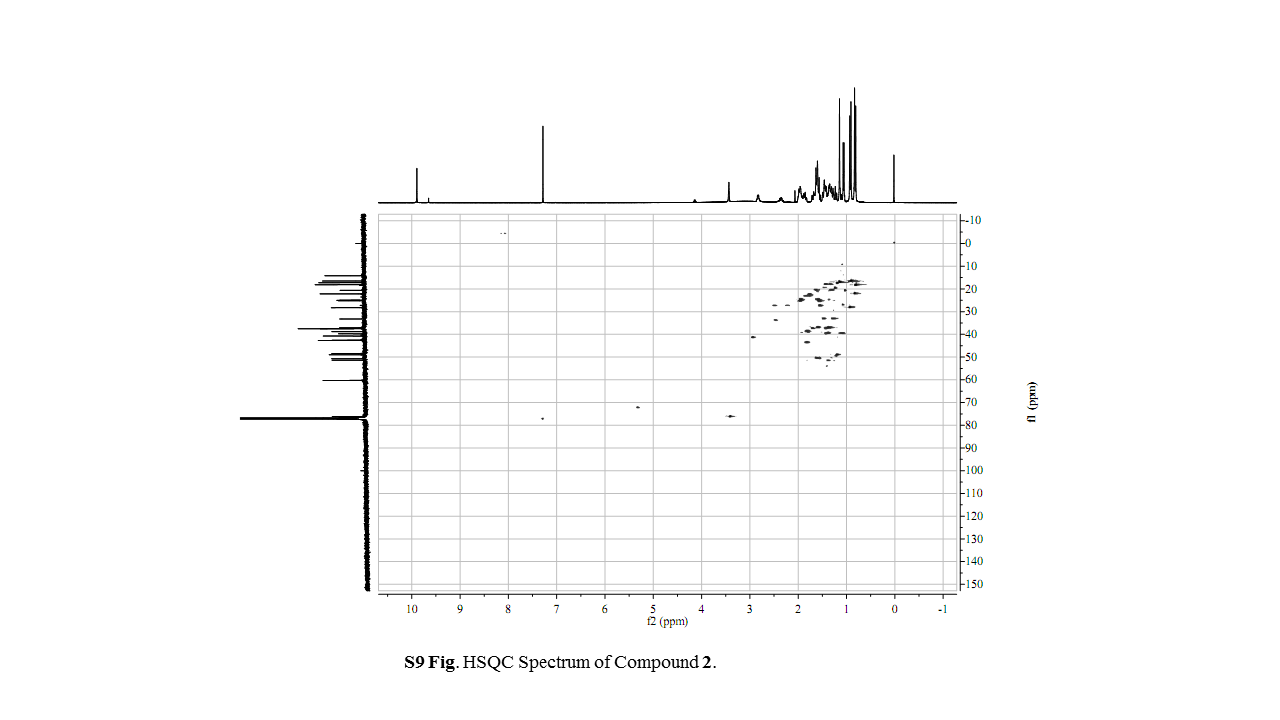

Supplement: S9 Fig — (TIF) [file pone.0175502.s009.TIF]

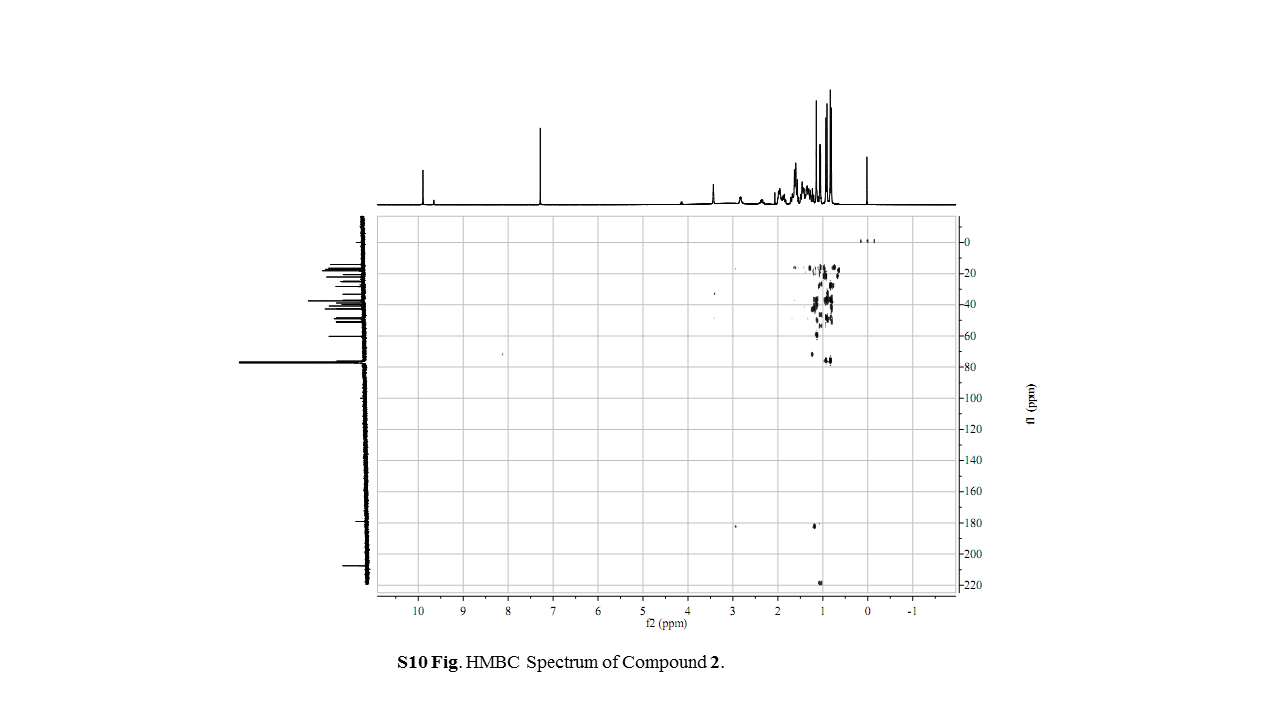

Supplement: S10 Fig — (TIF) [file pone.0175502.s010.TIF]

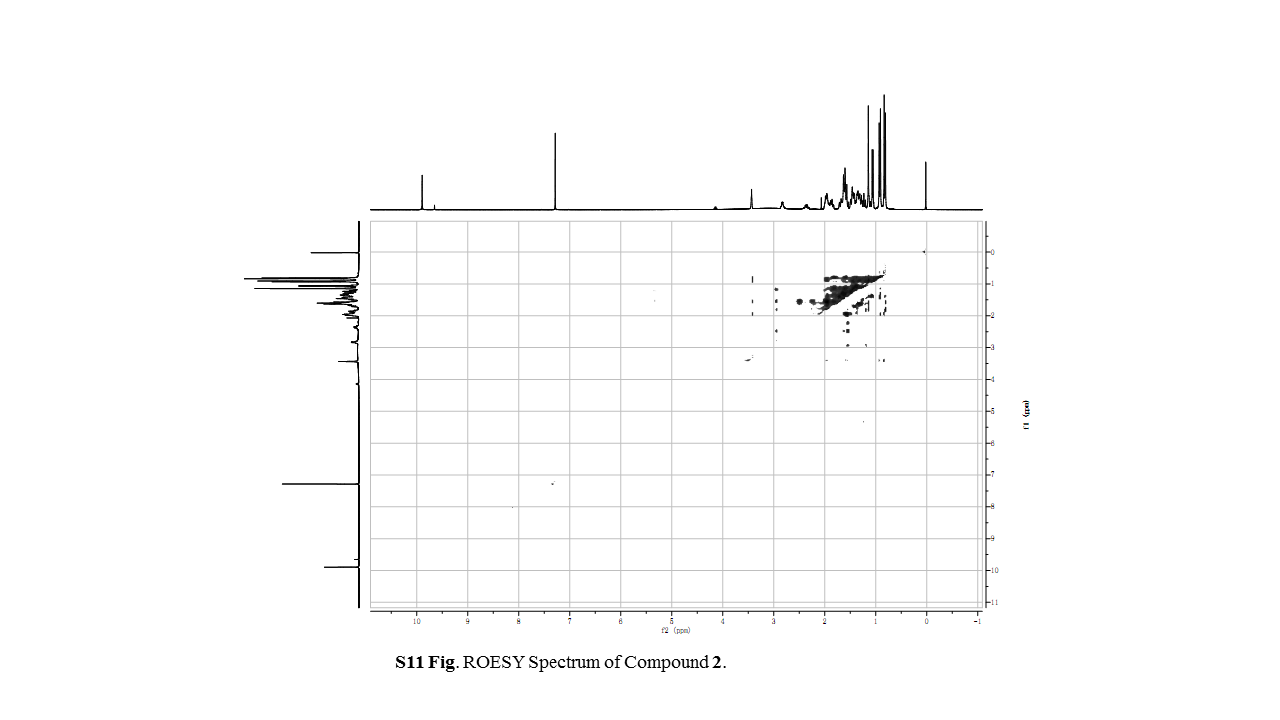

Supplement: S11 Fig — (TIF) [file pone.0175502.s011.TIF]

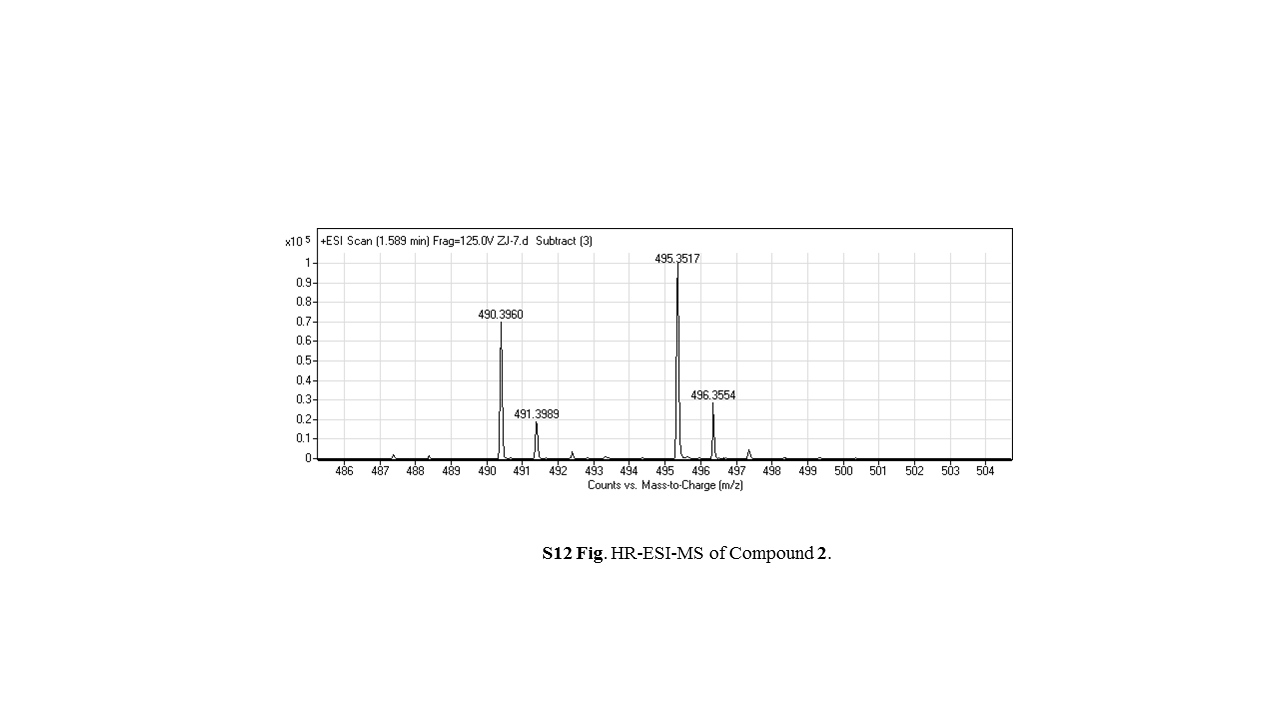

Supplement: S12 Fig — (TIF) [file pone.0175502.s012.TIF]

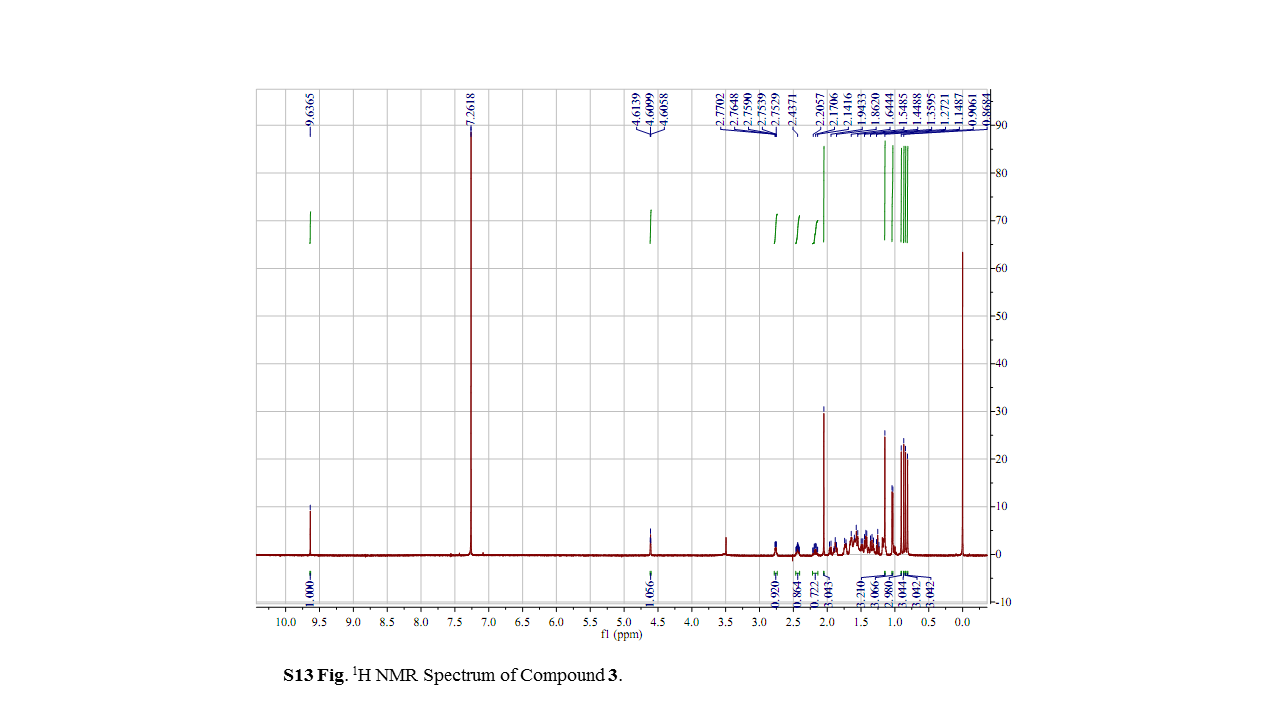

Supplement: S13 Fig — (TIF) [file pone.0175502.s013.TIF]

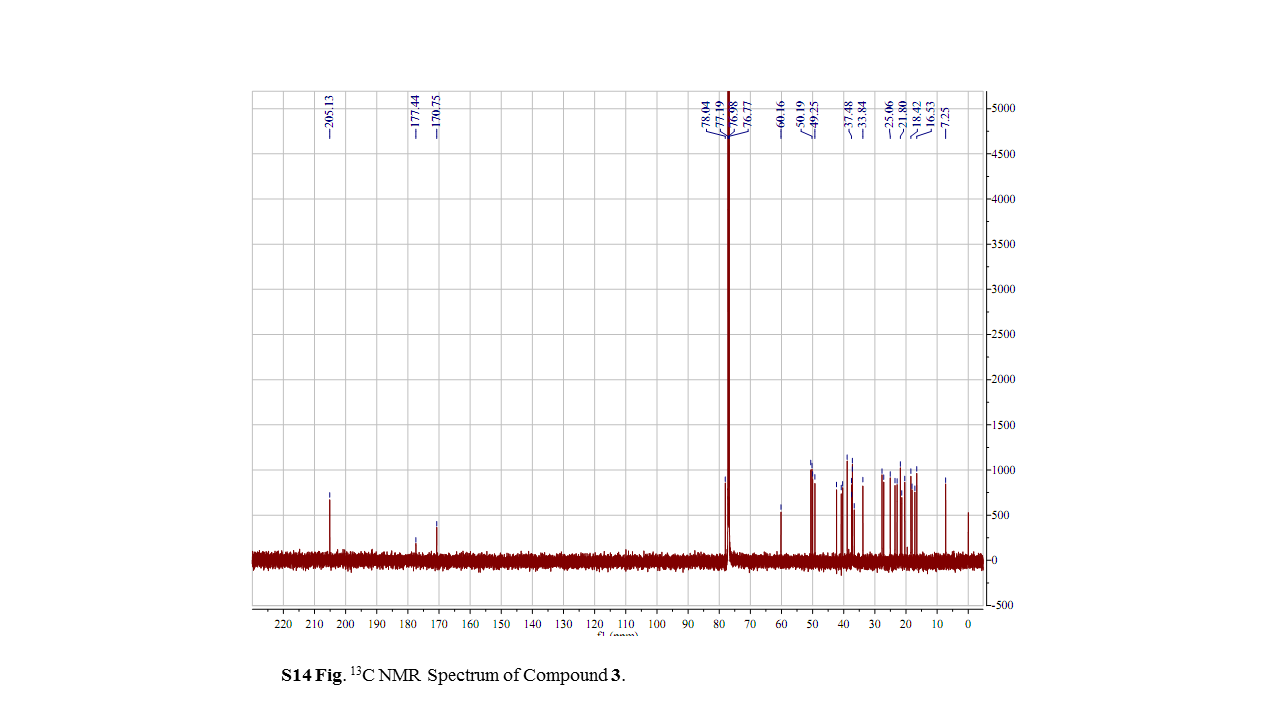

Supplement: S14 Fig — (TIF) [file pone.0175502.s014.TIF]

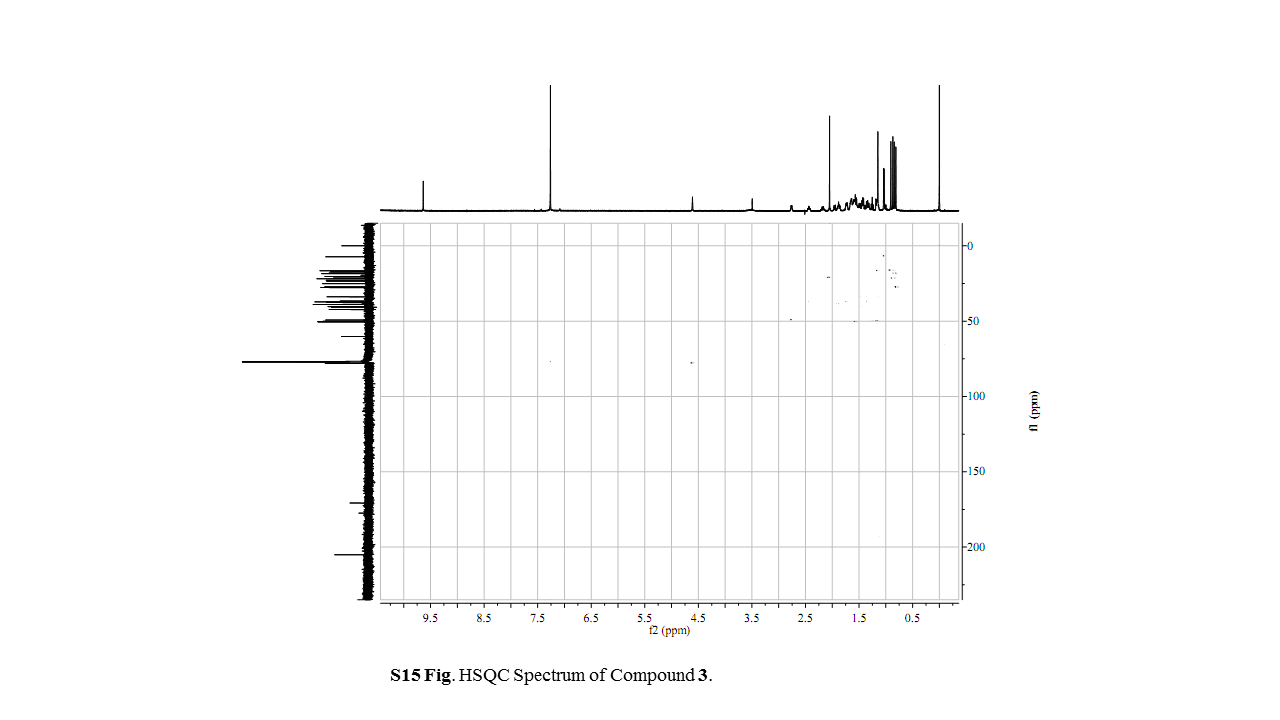

Supplement: S15 Fig — (TIF) [file pone.0175502.s015.TIF]

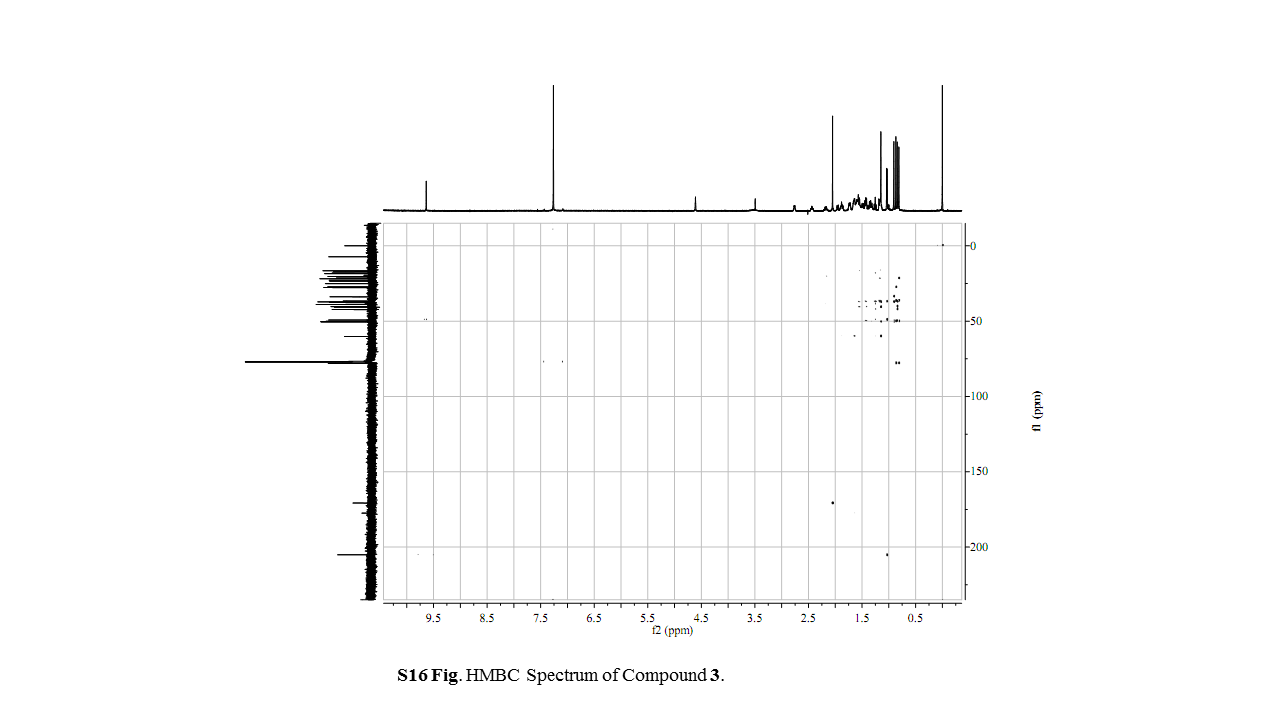

Supplement: S16 Fig — (TIF) [file pone.0175502.s016.TIF]

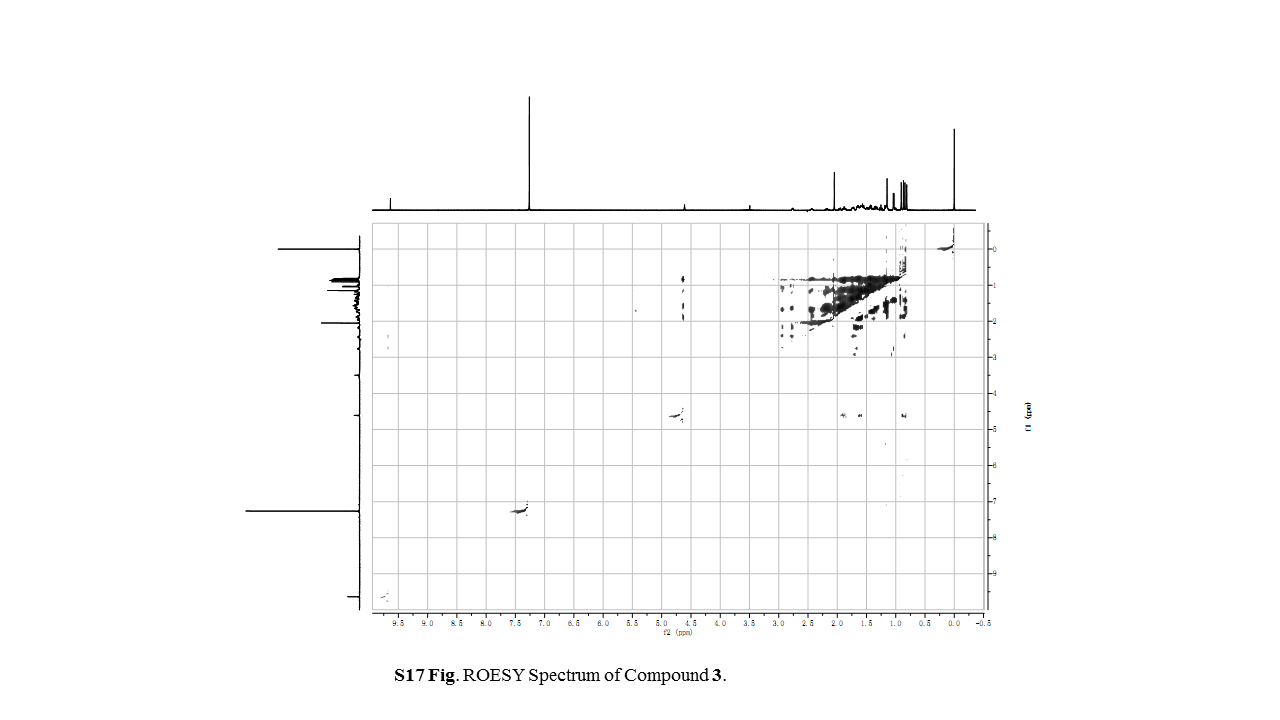

Supplement: S17 Fig — (TIF) [file pone.0175502.s017.TIF]

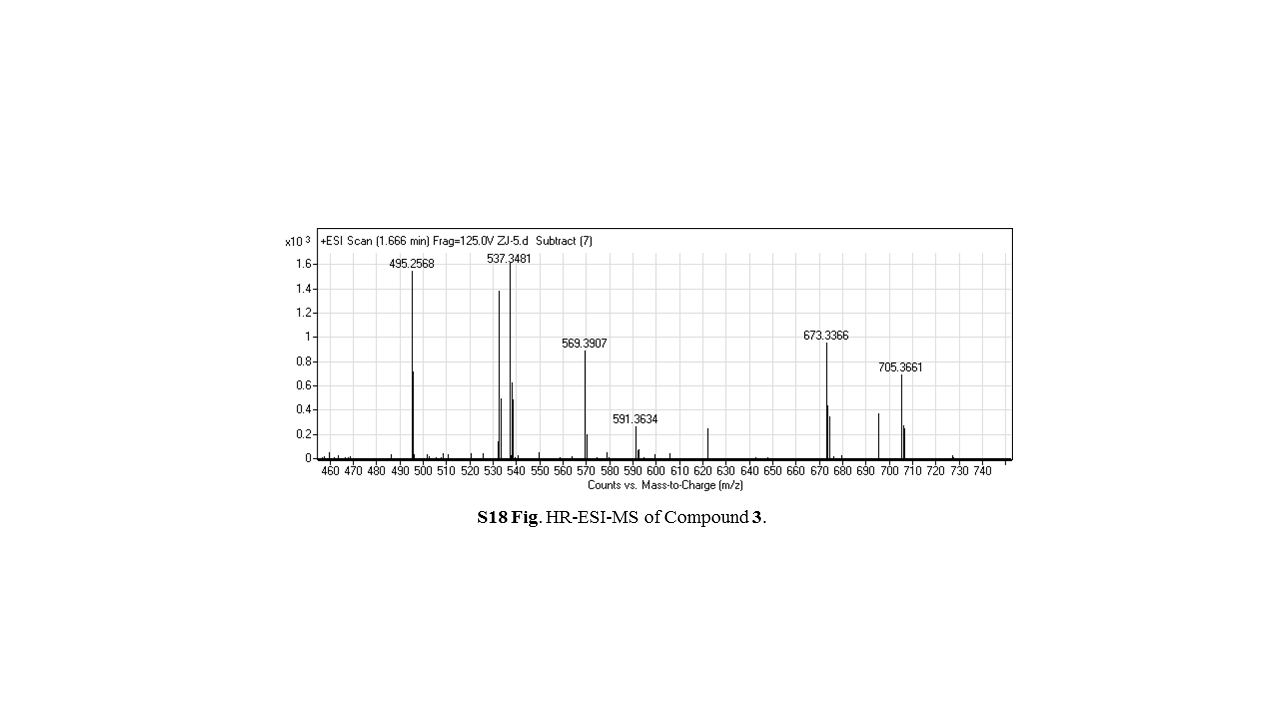

Supplement: S18 Fig — (TIF) [file pone.0175502.s018.TIF]

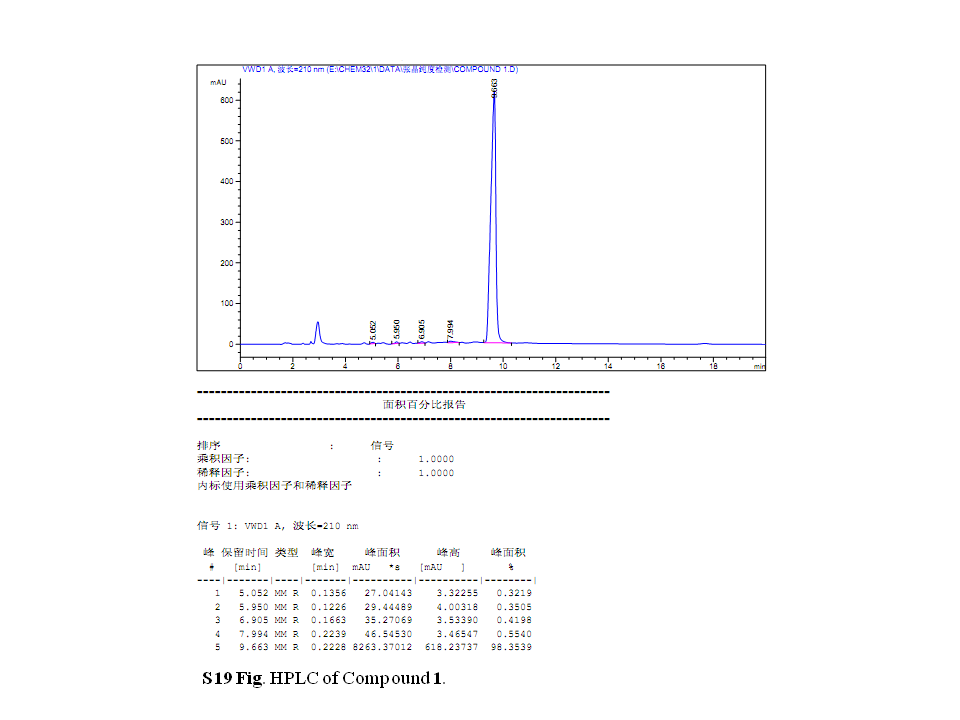

Supplement: S19 Fig — (TIF) [file pone.0175502.s019.tif]

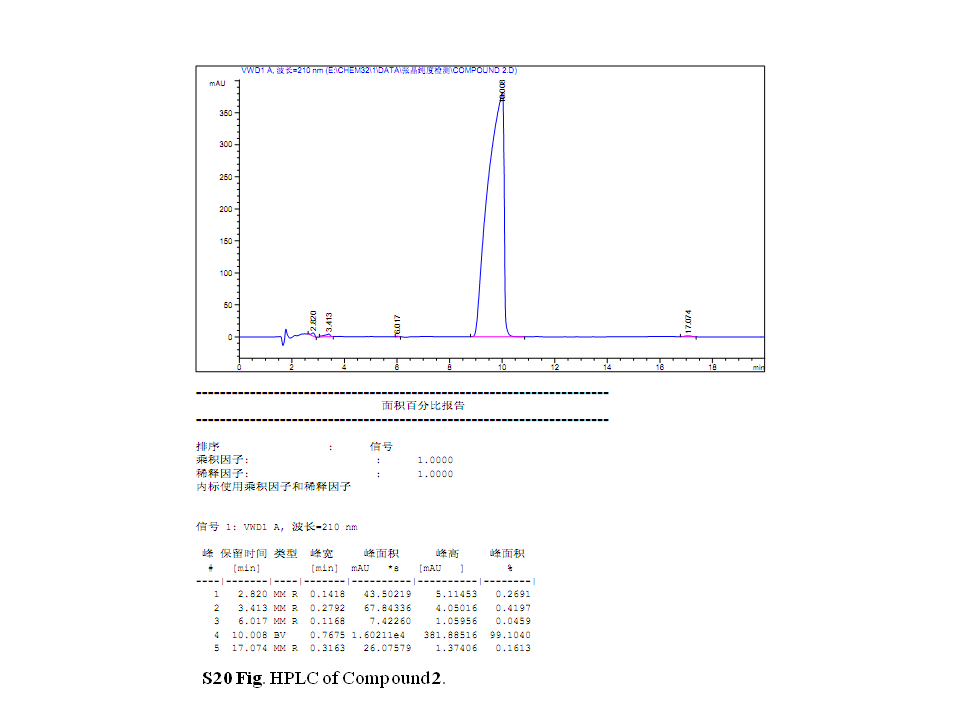

Supplement: S20 Fig — (TIF) [file pone.0175502.s020.tif]

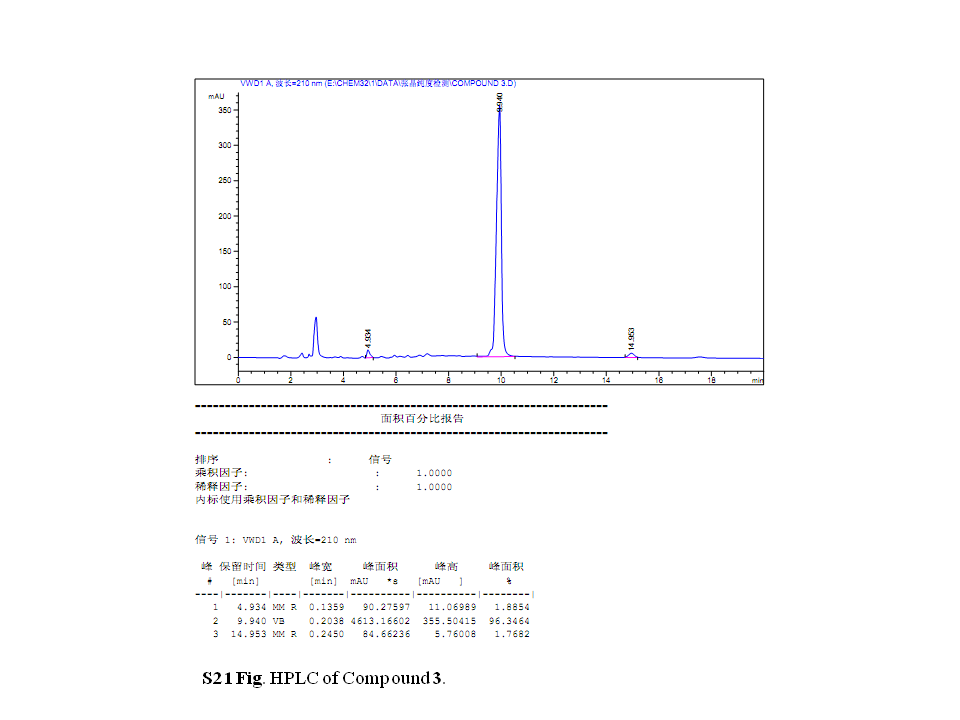

Supplement: S21 Fig — (TIF) [file pone.0175502.s021.tif]

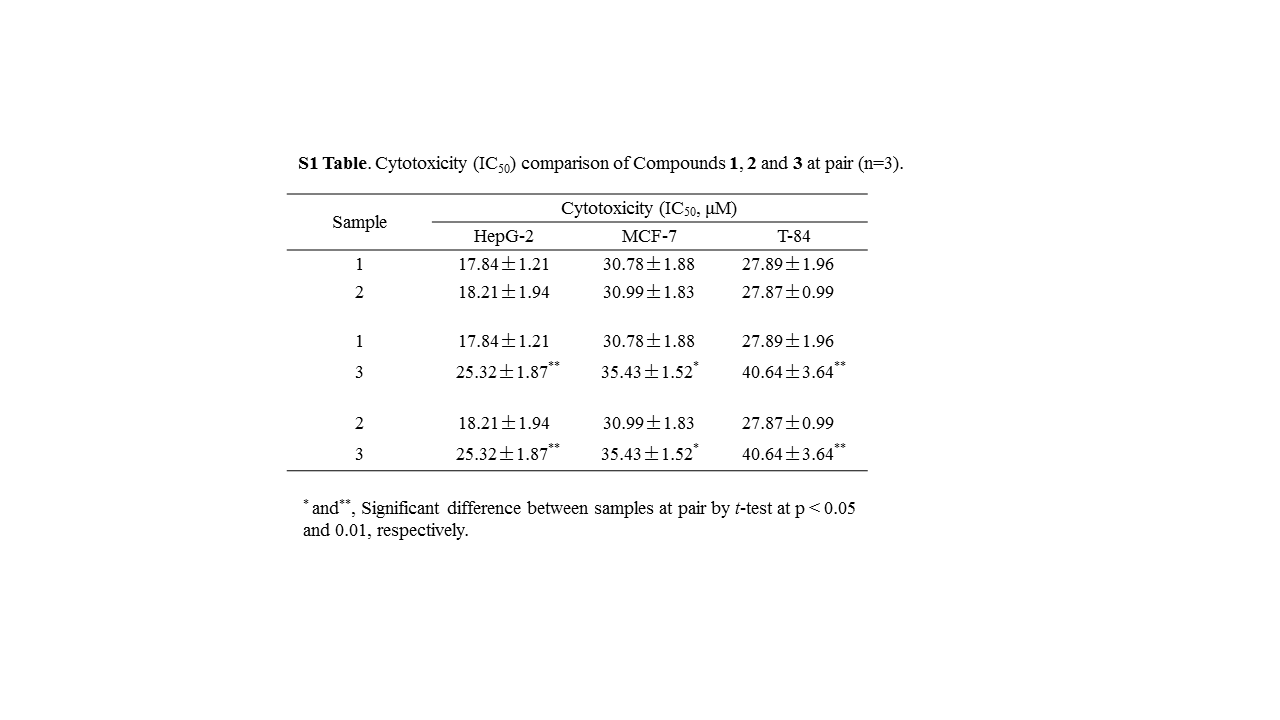

Supplement: S1 Table — (TIF) [file pone.0175502.s022.TIF]
